# Microenvironment-induced CREPT expression by cancer-derived small extracellular vesicles primes field cancerization

**DOI:** 10.7150/thno.87344

**Published:** 2024-01-01

**Authors:** Yuting Lin, Hanguo Jiang, Jun Li, Fangli Ren, Yinyin Wang, Ying Qiu, Jianghua Li, Mengdi Li, Ying Wang, Liu Yang, Yunhao Song, Huihui Jia, Wanli Zhai, Yanshen Kuang, Hanyang Yu, Wenyuan Zhu, Suling Liu, Eiichi Morii, Christian Ensinger, Charles David, Hanqiu Zheng, Jianguo Ji, Hongxia Wang, Zhijie Chang

**Affiliations:** 1 State Key Laboratory of Membrane Biology, School of Medicine, National Engineering Laboratory for Anti-tumor Therapeutics, Tsinghua University, Beijing 100084, China.; 2 Tsinghua-Peking Joint Center for Life Sciences, School of Medicine, School of Life Science, Tsinghua University, Beijing 100084, China.; 3 Department of General Surgery, General Hospital of PLA, Beijing 100700, China.; 4 State Key Laboratory of Protein and Plant Gene Research, School of Life Sciences, Peking University, Beijing 100871, China.; 5 Fudan University Shanghai Cancer Center and Institutes of Biomedical Sciences, Shanghai Medical College, Key Laboratory of Breast Cancer in Shanghai, Innovation Center for Cell Signaling Network, Cancer Institutes, Fudan University, Shanghai 200032, China.; 6 Department of Pathology, Osaka University Graduate School of Medicine, 2-2 Yamadaoka, Suita, Osaka 565-0871, Japan.; 7 Institute of Pathology, Medical University of Innsbruck, A-6020 Innsbruck, Austria.; 8 Department of Oncology, Shanghai General Hospital, Shanghai Jiao Tong University School of Medicine, Shanghai 200080, China.

**Keywords:** cancer-derived small extracellular vesicles, field cancerization, CREPT

## Abstract

**Rationale:** Cancer local recurrence increases the mortality of patients, and might be caused by field cancerization, a pre-malignant alteration of normal epithelial cells. It has been suggested that cancer-derived small extracellular vesicles (CDEs) may contribute to field cancerization, but the underlying mechanisms remain poorly understood. In this study, we aim to identify the key regulatory factors within recipient cells under the instigation of CDEs.

**Methods:** In vitro experiments were performed to demonstrate that CDEs promote the expression of CREPT in normal epithelial cells. TMT-based quantitative mass spectrometry was employed to investigate the proteomic differences between normal cells and tumor cells. Loss-of-function approaches by CRISPR-Cas9 system were used to assess the role of CREPT in CDEs-induced field cancerization. RNA-seq was performed to explore the genes regulated by CREPT during field cancerization.

**Results:** CDEs promote field cancerization by inducing the expression of CREPT in non-malignant epithelial cells through activating the ERK signaling pathway. Intriguingly, CDEs failed to induce field cancerization when CREPT was deleted, highlighting the importance of CREPT. Transcriptomic analyses revealed that CDEs elicited inflammatory responses, primarily through activation of the TNF signaling pathway. CREPT, in turn, regulates the transduction of downstream signals of TNF by modulating the expression of TNFR2 and PI3K, thereby promoting inflammation-to-cancer transition.

**Conclusion:** CREPT not only serves as a biomarker for field cancerization, but also emerges as a target for preventing the cancer local recurrence.

## Introduction

Cancer recurrence *in situ*, also acknowledged as the local recurrence of cancer, threatens the health of patients undergoing tumor resection, resulting in a decline in the survival rate of patients. Previous studies revealed that the local recurrence is related to the residual tumor 1 or adjacent tissues influenced by primary tumors 2-4. Several groups observed that certain ostensibly normal tissues adjacent to the tumor often showed alterations in phenotype and gene expression patterns 4-11. The phenotype alterations include an increased growth rate, decreased death rate or increased immune evasion in normal tissues, similar to the hallmarks of tumors 12, 13. Correspondingly, alteration of the gene expression patterns occurs through gene mutation, epigenetic modification and transcriptome shift. The altered cells in adjacent normal tissues are able to transform into malignant cells and may gain growth advantages and develop into neoplasms after tumor removal. This phenomenon has been observed since 1953 and referred as field cancerization 12. To date, it has been widely recognized that field cancerization occurs in normal tissues subjected to mutagenic insults from either external mutagens including chronic ultraviolet radiation, smoke and virus infections or the adjacent tumor-secreted factors. Several reports have suggested that field cancerization is due to chronic and local inflammations, as tumor-secreted inflammatory factors including IL-6, TNF-a and iNOS induced the oncogenic gene expression in the adjacent normal cells 14-16. However, it is still unclear how the adjacent primary tumor triggers field cancerization in the surrounding normal cells.

Recently, three groups reported that small extracellular vesicles secreted by cancer cells are able to prime the surrounding normal epithelial cells, contributing to field cancerization 17-19. Small extracellular vesicles with a diameter ranging from approximately 30 to 200 nm are recognized mainly to exosomes 20. As important mediators of cell-to-cell communications, small extracellular vesicles have drawn extensive attention in recent years due to their vital roles in the occurrence and development of tumors 21-24. Numerous studies have reported the important role of cancer-derived small extracellular vesicles (CDEs) in the tumor microenvironment, which affect the pathological activities of fibroblasts 25, endothelial cells 26, and immune cells 27, thereby regulating nutrient supply, angiogenesis, and immune escape. In addition, two studies demonstrated that CDEs induce field cancerization of normal epithelial cells 18, 28, indicating that CDEs are able to function as a type of carcinogenic insults. Further studies have raised a possibility of CDEs as triggers for field cancerization in prostate cancer and gastric cancer 17, 29. All the studies suggest that the normal epithelial cells adjacent to the cancer undergoing field cancerization are likely due to instigation by CDEs. However, the specific factors that play significant roles in this process remain unknown.

CREPT is an oncoprotein identified more than 20 years ago in our laboratory 30, 31. Our lab firstly reported that CREPT accelerates the transcription by promoting the chromatin loop formation 30. This protein is highly expressed in many tumors and negatively correlated to the survival rate for cancer patients, and possibly could be used as a prognosis marker 30. In recent years, our studies revealed that CREPT promotes the activation of cancer-related signaling pathways including Wnt 32 and STAT3 33, and also the cell cycle transition 34. Other studies confirmed the role of CREPT in the tumorigenesis and extended the function on tumor diagnosis in different cancers 31, 35-38. In this study, we report that CREPT is induced in non-malignant epithelial cells by CDEs. Strikingly, we found that deletion of CREPT impaired the CDEs-induced field cancerization. We propose that CREPT is a gatekeeper to switch on field cancerization in non-malignant cells in response to insults by CDEs.

## Results

### CREPT is upregulated in the cancerized field adjacent to the tumor

**Figure 1:**
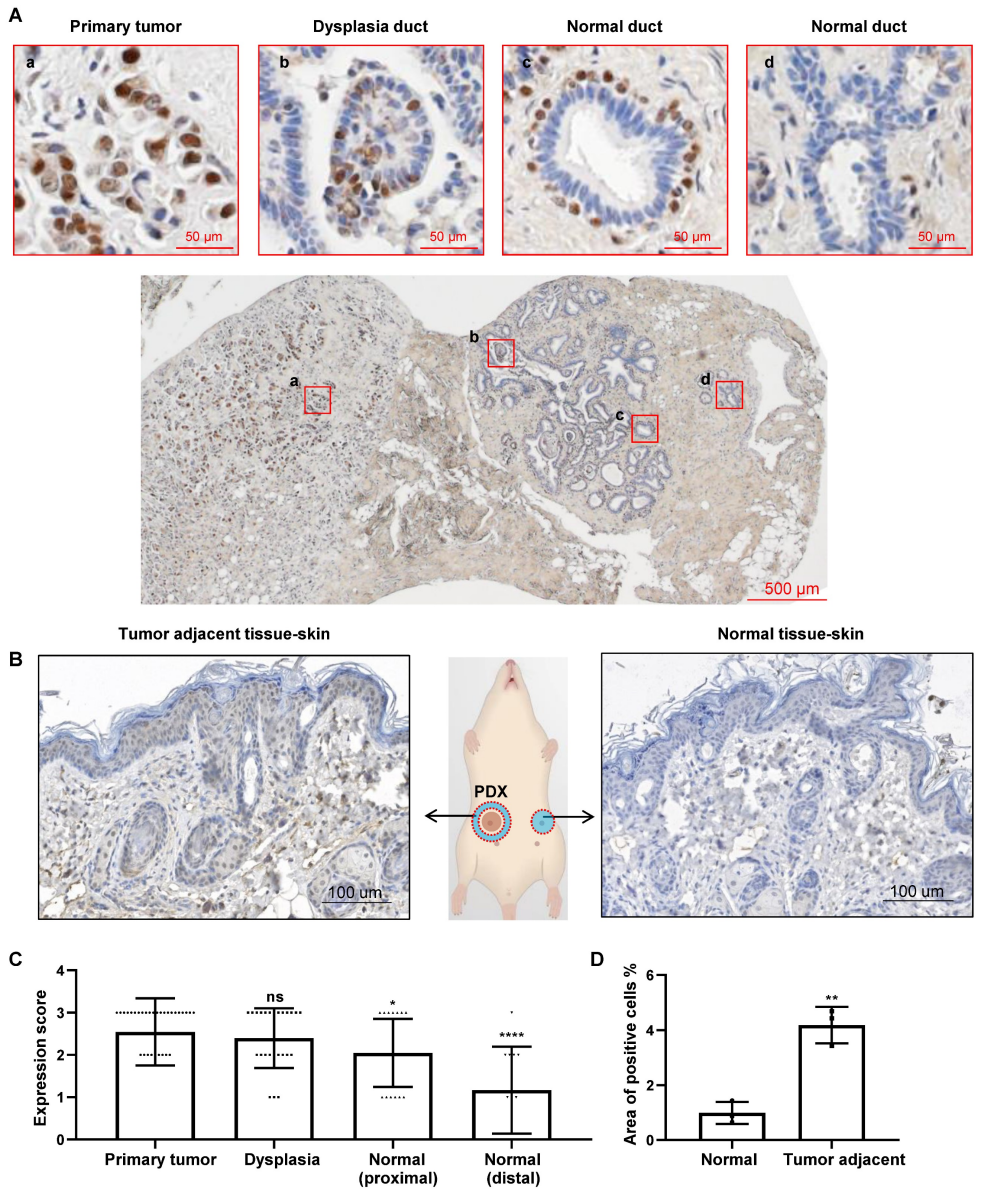
** CREPT upregulated in the cancerized field adjacent to the tumor.** (A) IHC staining of CREPT in human breast ductal carcinoma. CREPT is highly expressed in the primary tumor (a), and is induced in the epithelial cells in the dysplasia duct (b) and the myoepithelial cells of normal ducts (c), but not expressed in the distal normal ducts (d). (B) IHC staining of CREPT in mouse skin adjacent to PDX tumor and the skin in the opposite site without PDX tumor. (C) The statistics analysis of CREPT expression in primary tumors, dysplasia, proximal normal tissues and distal normal tissues. The expression scoring was determined based on the intensity of DAB staining, with a score of 3 assigned to strong staining, a score of 2 assigned to moderate staining, a score of 1 assigned to weak staining, and a score of 0 assigned to negative staining. (D) The statistics analysis of positive area of CREPT in mouse skin adjacent to PDX tumor and the skin in the opposite site without PDX tumor, n = 3.

Our previous study suggested that CREPT emerged in the paracancerous tissues 30. To observe the continuous distribution of CREPT expression from tumor to the adjacent tissue, we collected a number of slices containing primary tumors and the surrounding tissues. An immunohistochemical (IHC) staining experiment demonstrated that CREPT was highly expressed in the primary tumor cells in a breast ductal carcinoma patient (Figure 1Aa), consistent with our and other's previous observations 30, 39. Interestingly, we found that CREPT expression was restricted to certain cells in the adjacent tissue. In a section near to the primary tumor, CREPT was positive in several epithelial cells localized in a dysplastic duct, which was considered as a cancerized field (Figure 1Ab). In another section between the primary tumor and distal normal tissue, CREPT-positive cells appeared surrounding the negative epithelial cells in the normal duct (Figure 1Ac). These positive cells were easily identified as myoepithelial cells in the breast gland according to the histological structure. Furthermore, CREPT expression appeared completely negative in the distal normal tissue (Figure 1Ad).

All of these results indicated a distance-dependent expression pattern of CREPT in the breast tumor. Then, we conducted an immunostaining analysis on the slides of 33 breast cancer patients with positive CREPT expression from a total of 64 samples. Through the evaluation of CREPT staining in these 33 patients, we found that CREPT was highly expressed in the primary tumor tissues of 66.7% of the patients, consistent with our previous observations 30. Surprisingly, high expression of CREPT was also observed in the adjacent hyperplastic tissues of 52% of the patients, while CREPT expression was nearly absent in the distal normal tissues of 58.3% of the patients (Table 1). We conducted a statistical analysis of CREPT expression at different locations and found that the expression of CREPT indeed exhibited a distance-dependent pattern, with higher levels of CREPT expression in normal tissues closer to the tumor and lower levels in normal tissues further away (Figure 1C). The distance-dependent CREPT expression pattern was further confirmed in the cervical (Figure S1A), and colorectal (Figure S1B) carcinomas. All of the results resemble a feature of field cancerization in a distance-dependent manner relative to the primary tumor. In fact, we observed high expression of CREPT in many adjacent normal tissues near the tumor, suggesting a signal for pre-malignant lesions. Therefore, we conducted IHC experiments on breast hyperplastic tissues, and the results revealed that 9 out of 10 patients exhibited high expression of CREPT in their hyperplastic tissues (Figure S1C). Overall, these findings suggest the potential of CREPT as a biomarker for field cancerization.

In order to address whether the increased CREPT expression in non-malignant tissues is due to the primary tumor, we performed an IHC experiment in the adjacent skin tissues from mice subcutaneously inoculated with the patient-derived xenograft (PDX) breast tumors. The result showed that CREPT expression was elevated in the mouse skin near the human breast tumor (Figure 1B, left) but not in the opposite skin tissue without the PDX tumor (Figure 1B, right). We generated three PDX models of breast cancer, and observed that the expression level of CREPT in the adjacent skin tissue of the tumor was significantly higher compared to the normal skin tissue (Figure 1D). This result suggests that the human PDX breast tumor is able to induce CREPT expression in the normal mouse skin.

### CDEs promote CREPT expression in the non-malignant epithelial cells

As CDEs are reported to play a role in field cancerization 17 and have an ability to transform normal epithelial cells 28, we speculated that CDEs might be responsible for the induced CREPT expression. To this end, we determined to compare the interplay effect of two non-malignant epithelial cell lines, NMuMG (of murine origin) and MCF10A (of human origin), and two breast cancer cell lines, 4T1 (of murine origin) and MDA-MB-231 (of human origin). First, we co-cultured NMuMG cells with 4T1 cells in separate chambers, which only allows transmission of secreted factors between the chambers (Figure S2A, upper panel). The result showed that the expression level of CREPT was increased in NMuMG cells co-cultured with 4T1 cells (Figure S2A, lower panels, lane 3), suggesting that CREPT is induced by cancer cells. Interestingly, the addition of GW4869, an inhibitor of small extracellular vesicle secretion, repressed the upregulation of CREPT (Figure S2A, lower panels, lane 4). This result suggested that it might be CDEs that trigger CREPT expression in the non-malignant epithelial cells.

**Figure 2:**
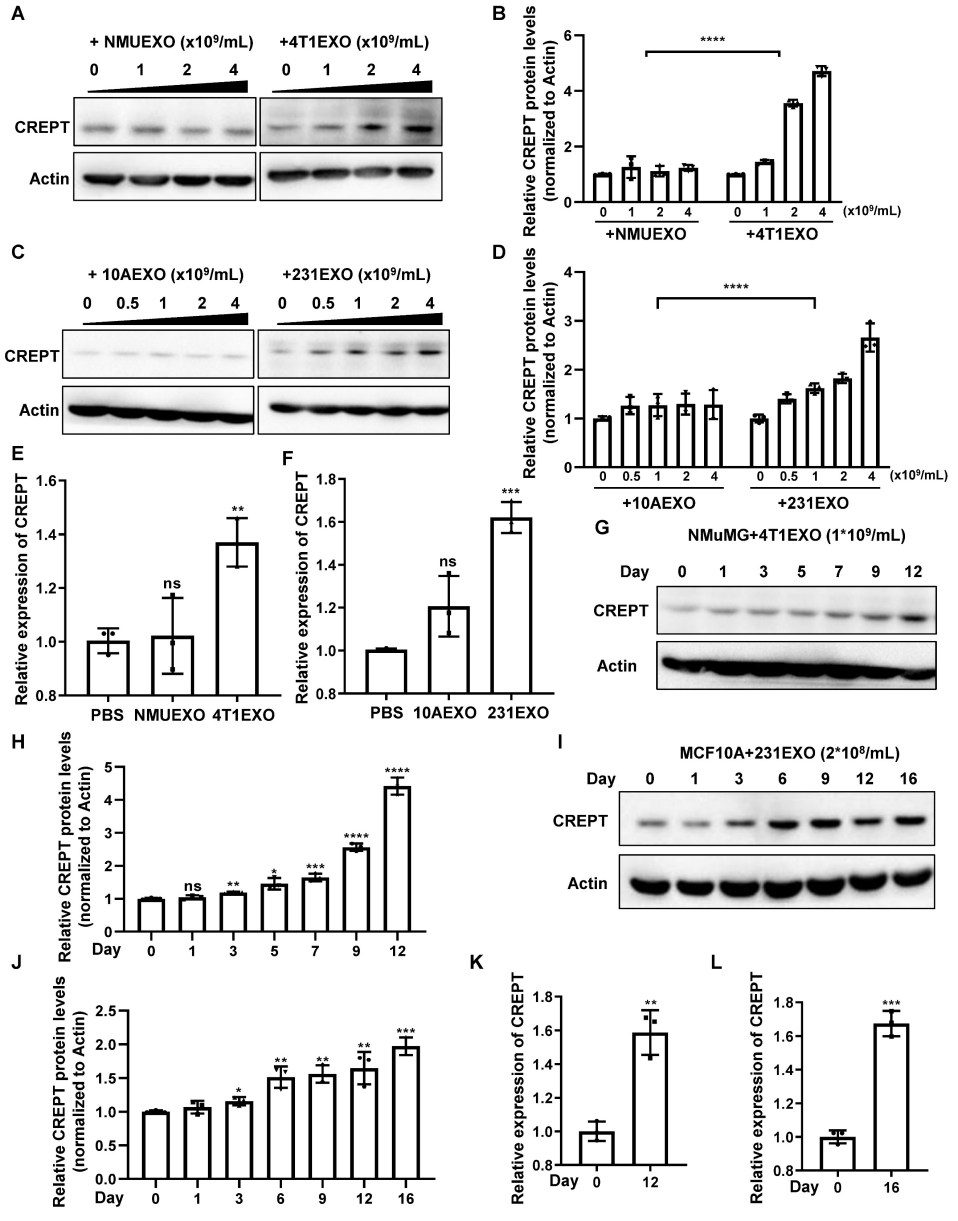
** CDEs induce CREPT expression in non-malignant epithelial cells.** (A) Western blot of CREPT in protein extracts of NMuMG cells treated for 24 h with NMUEXO or 4T1EXO in the concentration of 0, 1, 2 and 4 × 10^9^ particles/mL. The statistics analysis of CREPT protein levels was present (B), n = 3. (C) Western blot of CREPT in protein extracts of MCF10A cells treated for 24 h with 10AEXO or 231EXO in the concentration of 0, 0.5, 1, 2 and 4 × 10^9^ particles/mL. The statistics analysis of CREPT protein levels was present (D), n = 3. (E) The mRNA level of CREPT was quantified by qPCR of NMuMG cells treated for 24 h with PBS, NMUEXO, or 4T1EXO. (F) The mRNA level of CREPT was quantified by qPCR of MCF10A cells treated for 24 h with PBS, 10AEXO or 231EXO. (G) Western blot of CREPT in protein extracts of NMuMG cells treated for 0, 1, 3, 5, 7, 9 and 12 days with 4T1EXO in the concentration of 1 × 10^9^ particles/mL. The statistics analysis of CREPT protein levels was present (H), n = 3. (I) Western blot of CREPT in protein extracts of MCF10A cells treated for 0, 1, 3, 6, 9, 12, and 16 days with 231EXO in the concentration of 2 × 10^8^ particles/mL. The statistics analysis of CREPT protein levels was present (J), n = 3. (K) The mRNA level of CREPT was quantified by qPCR of NMuMG cells treated with 4T1EXO for 12 days. (L) The mRNA level of CREPT was quantified by qPCR of MCF10A cells treated with 231EXO for 16 days.

To demonstrate the effects of CDEs on non-malignant epithelial cells, we isolated small extracellular vesicles from the aforementioned four cell types, which were named NMUEXO, 10AEXO, 4T1EXO, and 231EXO. The NMUEXO and 10AEXO represent small extracellular vesicles derived from non-malignant epithelial cells (NDEs), while the 4T1EXO and 231EXO represent CDEs. A transmission electron microscopy (TEM) analysis verified the classic teacup-like structure of the isolated small extracellular vesicles (Figure S2B), and a nanoparticle tracking analysis revealed the particle size to be between 30 and 200 nm (Figure S2C). Western blot results indicated that the small extracellular vesicles we isolated contained positive markers CD9, CD63, and CD81, but lacked the negative marker Calnexin (Figure S2D). We then treated MCF10A cells with different concentrations of 10AEXO and 231EXO, and simultaneously treated NMuMG cells with NMUEXO and 4T1EXO. The result showed that the protein level of CREPT was increased in NMuMG (Figure 2A-B) and MCF10A cells (Figure 2C-D) treated with CDEs in a dose dependent manner, but not with NDEs (Figure 2A-C, left panel). Consistently, the mRNA level of CREPT was also increased by CDEs, but not by NDEs (Figure 2E-F). Furthermore, we observed that CDEs secreted by colon cancer cell SW620 (named SW620EXO) also induced CREPT expression in the normal colon epithelial cell NCM460, while NDEs from NCM60 (named NCM460EXO) had no effect (Figure S2E). Simultaneously, we observed that the CREPT expression in NMuMG and MCF10A cells induced by CDEs was increased in a time-dependent manner (Figure 2G-L). All these results suggest that CDEs, but not NDEs, are able to induce the expression of CREPT in the non-malignant epithelial cells.

### CDEs promote CREPT expression by activating ERK

**Figure 3:**
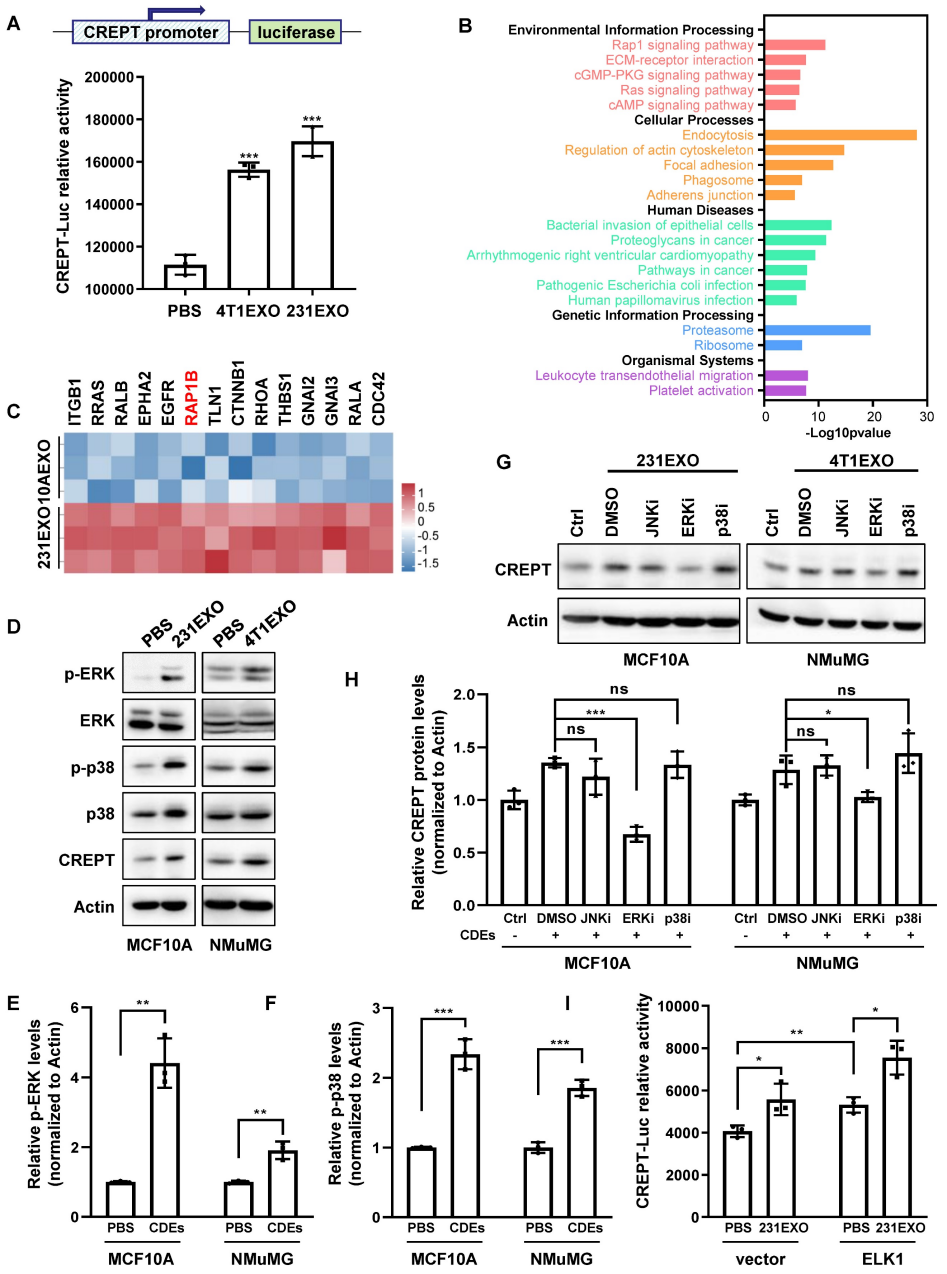
** CDEs induce CREPT expression through ERK activation.** (A) The luciferase reporter activity in CHO cells transfected with a luciferase gene driven by CREPT-promoter and treated with PBS, 4T1EXO or 231EXO. (B) KEGG analysis of the 195 differentially upregulated proteins (p-value < 0.05) in 231EXO compared with MCF10AEXO. The -Log10(p-value) is indicated. (C) The heatmap of relative levels of 14 proteins associated with RAP1 signaling pathway enriched in 231EXO. (D) Western blot of p-ERK, ERK, p-p38, p38 and CREPT in the protein extracts of MCF10A cells treated with PBS or 231EXO, and in protein extracts of NMuMG cells treated with PBS or 4T1EXO. The statistics analysis of p-ERK and p-p38 levels was present (E-F), n = 3. (G) Western blot of CREPT in protein extracts of MCF10A cells or NMuMG cells treated with PBS plus DMSO or treated with 231EXO plus DMSO, JNK inhibitor (SP600125, 10 µM), ERK inhibitor (SCH772984, 5 µM) or p38 inhibitor (SB203580, 10 µM). All inhibitors were dissolved in DMSO as the solvent. The statistics analysis of CREPT protein levels was present (H), n = 3. (I) The luciferase reporter activity in 293T cells transfected with the plasmid carrying a luciferase gene driven by CREPT-promoter plus the vector of pcDNA3.1 or pcDNA3.1/flag-ELK1 treated with PBS or 231EXO.

We next investigated how CDEs elevate CREPT expression in non-malignant epithelial cells. First, we questioned if CDEs contained the CREPT protein. A Western blot analysis showed that CREPT was undetectable in 4T1EXO and 231EXO (Figure S3A), as well as B16EXO (Figure S3B), although Vesiclepedia database (http://www.microvesicles.org) reported the presence of the CREPT protein in small extracellular vesicles from several cancer cell lines. Thus, we speculated that CDEs-induced CREPT expression could be due to transcriptional regulation. To test this hypothesis, we generated a cell line by stably expressing a CREPT promoter-driven luciferase reporter gene based on CHO cells. A luciferase reporter assay showed that CDEs boosted the CREPT promoter-driven luciferase activity significantly (Figure 3A and Figure S3C). These results suggest that CDEs induce the expression of endogenous CREPT in the non-malignant epithelial cells at the transcriptional level.

To uncover how CDEs regulate CREPT expression, we investigated the proteomic difference between CDEs and NDEs. We analyzed the protein profiles of SW620EXO and NCM460EXO from our quantitative mass spectrometry experiments and that of 231EXO and 10AEXO from published data 40. The results showed that 631 proteins were preferably abundant in SW620EXO compared with NCM460EXO (Figure S3D and Supplementary table 2), and 195 proteins were abundant in 231EXO compared with 10AEXO (p-value < 0.05). Interestingly, these differentially expressed proteins were enriched with the components of RAP1 signaling pathway (Figure 3B-C and Figure S3E-F). As the downstream effector events of RAP1B are ERK and p38 phosphorylation 41, we examined whether CDEs are able to activate these pathways. A Western blot analysis showed that the phosphorylation of both ERK and p38 was significantly increased in non-malignant epithelial cells after the addition of CDEs (Figure 3D-F). Of note, the CREPT protein level was elevated, accompanied with an increased level of phosphorylated ERK and p38 (Figure 3D). Intriguingly, CREPT expression was decreased to the basal level in CDEs-treated NMuMG and MCF10A cells only when ERK inhibitor, SCH772984, was added, but remained unchanged in the presence of p38 and JNK inhibitors (SB203580 and SP600125) (Figure 3G-H). Simultaneously, the expression level of CREPT also decreased in 4T1 cells with the addition of ERK inhibitor (Figure S3G). These results suggest that CDEs promote CREPT expression via activation of ERK. As ELK1 is a canonical downstream transcription factor of ERK, we examined the response to ELK1 on the reporter in the presence of CDEs. The result showed that over-expression of ELK1 promoted the CREPT promoter activity, which was further enhanced by the addition of CDEs (Figure 3I). Furthermore, we observed that ELK1 occupied the CREPT promoter of different cell lines (Figure S3H), as analyzed using the Cistrome Data Browser 42. All the results suggest that CDEs promote CREPT expression in the non-malignant epithelial cells by activating ERK.

### Over-expression of CREPT enhances the proliferation of non-malignant epithelial cells

**Figure 4:**
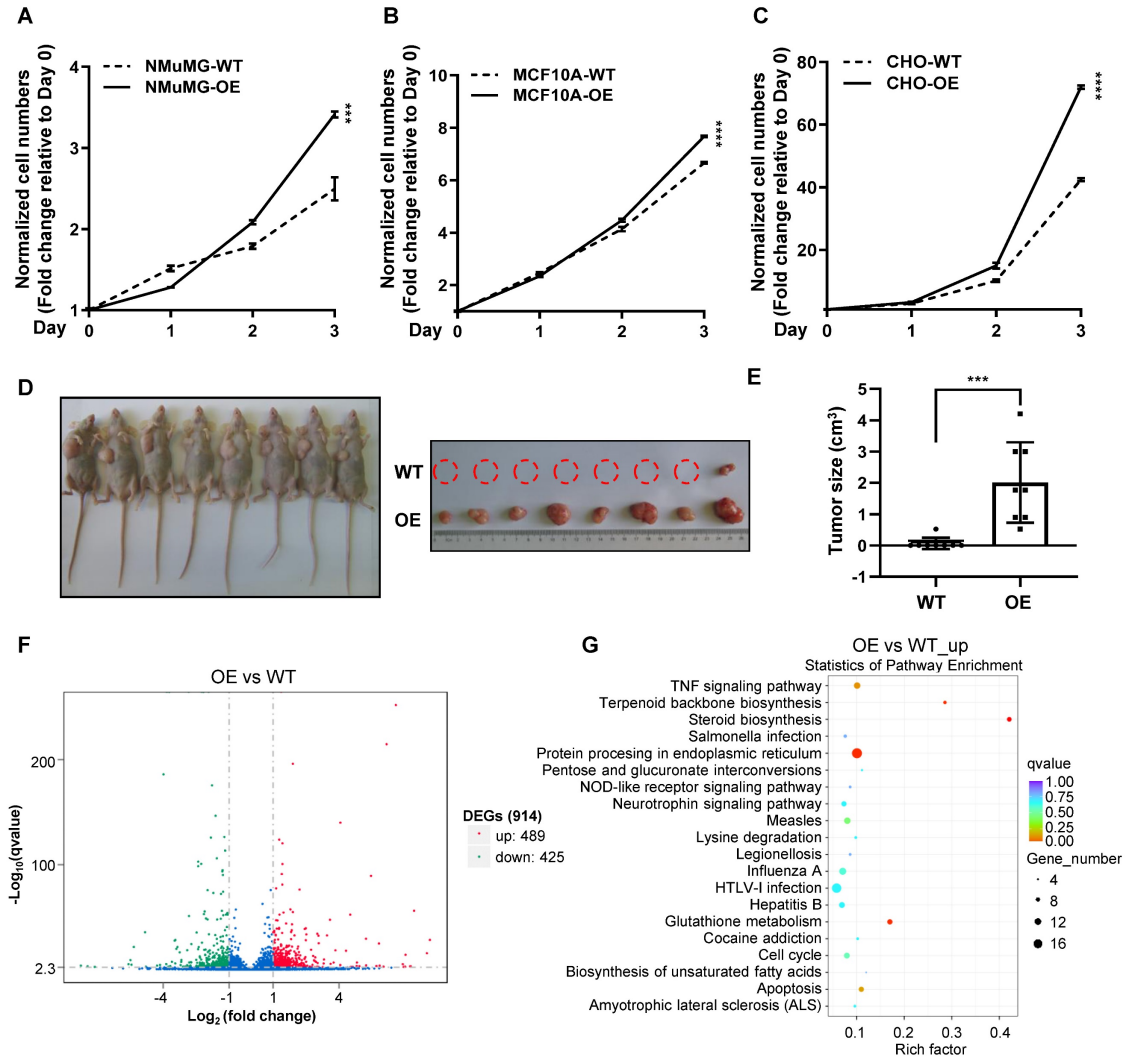
** Over-expression of CREPT enhances the proliferation of non-malignant epithelial cells.** CCK-8 assay over 3 days for NMuMG CREPT-WT/OE cells (A), MCF10A CREPT-WT/OE cells (B), and CHO CREPT-WT/OE cells (C), n = 3. The cell number indicated by OD450 is normalized to day 0. (D) The photo of the formed tumors (left photo) of CHO CREPT-WT/ OE cells injected subcutaneously into the nude mice for 5 weeks (n = 8 per group). CHO-OE cells were inoculated on the left side of nude mice, while CHO-WT cells were inoculated on the right side (right photo). (E) The statistical results of tumor size in Figure 4D, n = 8. (F) The volcano plot of Differentially Expressed Genes (DEGs) after CREPT over-expression in CHO. Green dots indicate down-regulation genes, red dots indicate up-regulation genes. (G) The bubble plot of KEGG enrichment analysis of up-regulated genes after CREPT over-expression in CHO.

To examine whether CREPT upregulation could promote the transition of normal epithelial cells into cancerized cells, we generated CREPT over-expression and knock-out cell lines based on NMuMG, MCF10A and CHO cells (Figure S4A-C). The results showed that over-expression of CREPT dramatically increased the proliferation rates of these non-malignant epithelial cells (Figure 4A-C). Intriguingly, we observed that the over-expression of CREPT transformed CHO cells, leading to their gained ability to form tumors in nude mice. (Figure 4D-E). Since CREPT has been reported to regulate transcription, we analyzed the transcriptome alteration upon CREPT over-expression or deletion. An RNA-seq analysis showed that over-expression of CREPT resulted in 489 genes upregulated based on the transcriptome of the CHO cells (Figure 4F and Supplementary table 3). Since CREPT acts as a co-activator of transcription factors 30, 32, 33, we focused on the positively regulated genes. A KEGG analysis revealed that these CREPT up-regulated genes were enriched in the inflammation-related signaling pathway like TNF signaling pathway (Figure 4G). Simultaneously, we observed that the deletion of CREPT appeared to affect the expression of genes related to the DNA damage repair and metabolism (Figure S4D-E). Taken together, these results suggest that CREPT over-expression promotes proliferation and even malignant transformation of non-malignant epithelial cells, and causes dramatic changes in the transcriptome.

### CREPT is required for the CDEs-induced field cancerization

Previous studies have shown that CDEs are involved in the field cancerization of tumor-adjacent tissues 17 and able to transform non-malignant epithelial cells 28. These cancerized cells showed an accelerated proliferation and enhanced clone formation ability. To verify the ability of CDEs to promote cancerization, we performed proliferation experiments with non-malignant epithelial cells incubated with CDEs for different time periods. The result showed that the non-malignant epithelial cells subject to CDEs treatment for increased times maintained increased proliferation rates even after CDEs withdrawal (Figure S5A). This result suggests that CDEs-treated non-malignant epithelial cells were altered towards a preference for accelerated proliferation, a feature of field cancerization.

**Figure 5:**
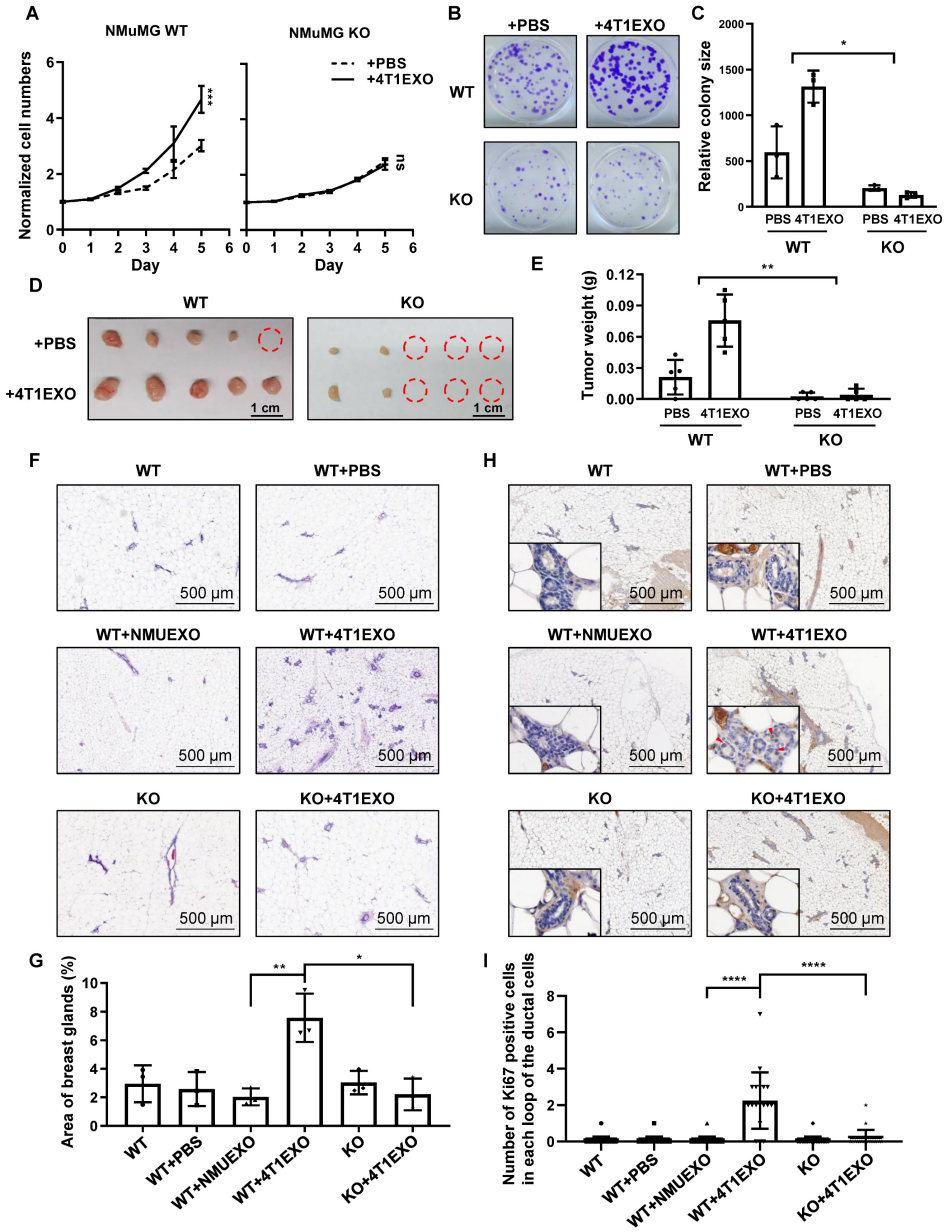
** CREPT is required for cancerization induced by CDEs.** (A) CCK-8 assay during 5 days of NMuMG CREPT-WT/ KO cells treated or untreated with 4T1EXO for 2 weeks, n = 3. The cell number indicated by OD450 is normalized by the value of day 0. (B) Representative images of cells stained with Crystal Violet showing colony formation from NMuMG CREPT-WT and CREPT-KO treated with PBS or 4T1EXO for two weeks. (C) Quantification of colony formation assay (B), the data shows the average size of each group. (D) The photo of the formed tumors from nude mice inoculated with NMuMG CREPT- WT/ KO cells treated or untreated with 4T1EXO for 10 weeks. These cells were orthotopically injected into the mammary pads of nude mice (n = 5 per group). (E) The statistical results of tumor weight in Figure 5D, n = 5. (F) The hematoxylin-eosin (HE) staining of the mammary gland in wild-type mice after the injection of PBS, NMUEXO or 4T1EXO in mammary fat pad for 14 days and the mammary gland in the mice with mammary specific-knockout of CREPT after the injection of 4T1EXO in mammary fat pad for 14 days. (G) The statistical results of Figure 5F, n = 3. (H) IHC staining of Ki67 in the corresponding breast tissue in Figure 5F. (I) The statistical results of Figure 5H, n = 20.

Next, we sought to address whether CREPT is critical for field cancerization. A CREPT deletion cell line was used to investigate the responses under the condition of preventing CDEs-induced CREPT elevation. The result showed that NMuMG cells failed to respond to the 4T1EXO treatment at proliferation (Figure 5A), colony formation (Figure 5B-C) and tumorigenicity (Figure 5D-E), when CREPT was deleted. Similarly, we observed that CDEs induced the colony formation of MCF10A, while CREPT deletion hampered this effect (Figure S5B-C). These results suggest that CREPT is required for the CDEs-induced field cancerization.

To further determine the role of CREPT in the CDEs-induced field cancerization *in vivo*, we generated the whole-body and mammary-specific *CREPT*-deletion mice. We injected small extracellular vesicles into the mammary fat pad of the wild-type and mammary-specific *CREPT*-deletion mice, once every two days for 14 consecutive days. Strikingly, 4T1EXO promoted mammary gland neogenesis in wild-type mice, while PBS or NMUEXO failed to do so. However, 4T1EXO failed to induce mammary gland neogenesis in the mammary-specific *CREPT*-deletion mice (Figure 5F-G). To verify the cancerization effect of CDEs on normal tissues, we performed IHC staining using Ki67, a marker of proliferation. The result showed that CDEs promoted the expression of Ki67 in the normal mammary glands of the wild-type mice, but not in the *CREPT*-deletion mammary glands (Figure 5H-I). In another group, we injected B16EXO subcutaneously in wild-type and whole-body *CREPT*-deletion mice. Simultaneously we observed that CREPT deletion blocked the hair follicle neogenesis and Ki67 elevation induced by B16EXO (Figure S5D-G). These results suggest that the CDEs-induced cancerization relies on the CREPT expression.

### CREPT-mediated TNF signaling promotes the CDEs-induced field cancerization

**Figure 6:**
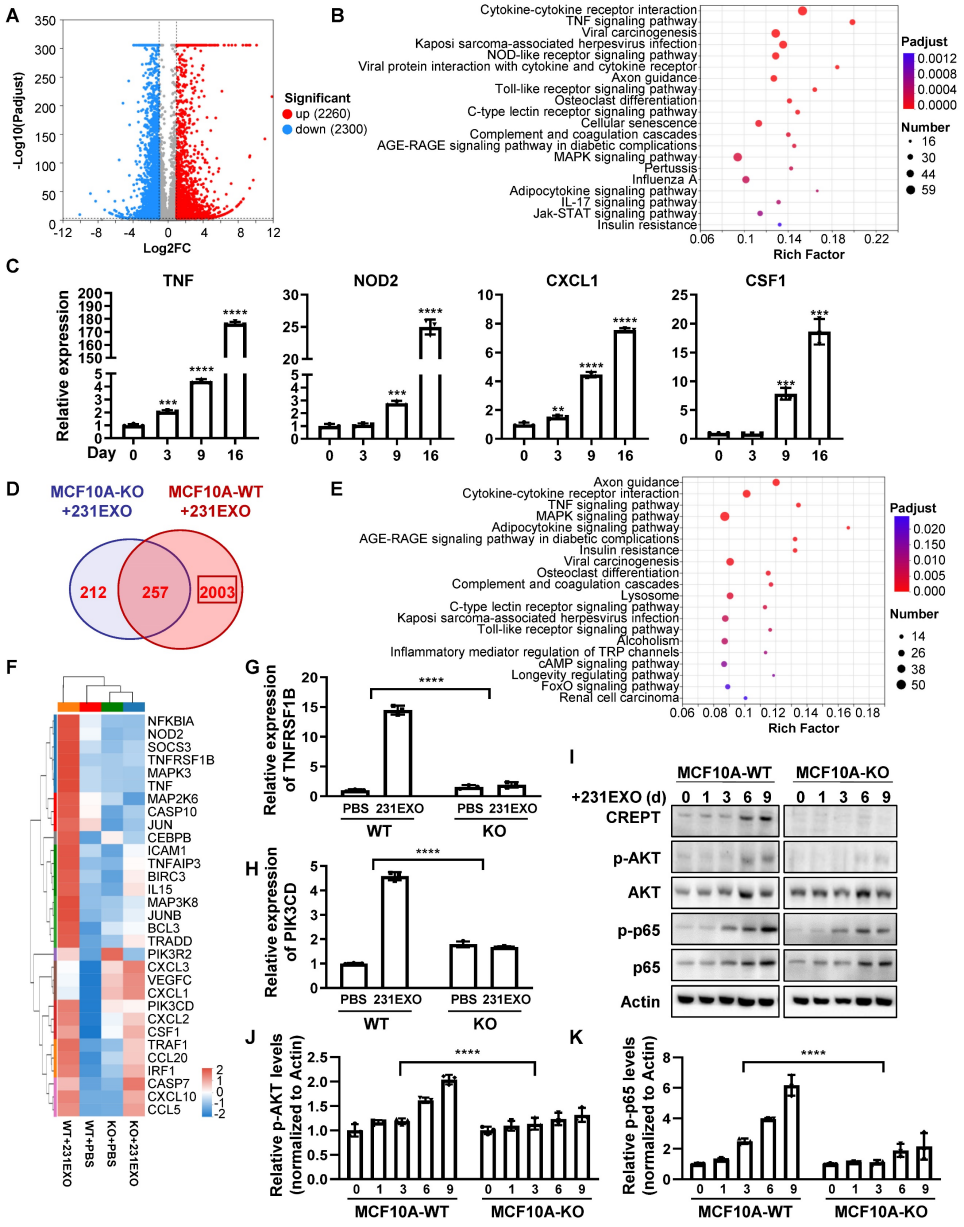
** CREPT-mediated TNFR2 signaling promotes field cancerization induced by CDEs.** (A) The volcano plot of the differentially expressed genes in MCF10A cells treated with 231EXO compared with untreated MCF10A cells. The blue dots show the significantly (fold change < 0.5, p-value < 0.001) down-regulated genes and the red dots show the significantly (fold change > 2, p-value < 0.001) up-regulated genes. (B) KEGG analysis of the differentially upregulated genes between untreated MCF10A cells and MCF10A cells treated with 231EXO for 2 weeks. The input gene number, p-value and rich factor of each signaling pathway are indicated. (C) The verification of the inflammation-related-mRNA levels of TNF, NOD2, CXCL1 and CSF1 by qPCR in MCF10A cells treated with 231EXO on day 0, 3, 9 and 16. (D) The Venn diagram shows the differentially expressed gene number in MCF10A WT/ KO cells treated with/ without 231EXO. (E) KEGG analysis of genes that were significantly upregulated in MCF10A CREPT-WT cells but not significantly up-regulated in MCF10A CREPT-KO cells. The input gene number, p-value and rich factor of each signaling pathway are indicated. (F) Heatmap of the mRNA levels of genes related to TNF signaling pathway in MCF10A CREPT-WT and CREPT-KO cells treated with PBS or 231EXO. The mRNA levels of TNFRSF1B (G) and PIK3CD (H) were quantified by qPCR of MCF10A WT/ KO cells treated with/ without 231EXO. (I) Western blot of CREPT, p-AKT, AKT, p-p65 and p65 in protein extracts of MCF10A CREPT-WT and CREPT-KO cells treated with 231EXO for 0, 1, 3, 6 and 9 days. The statistics analysis of p-AKT and p-p65 levels was present (J-K), n = 3.

To explore the underlying mechanism of how CREPT promotes the CDEs-induced field cancerization, we performed RNA-Seq analyses for normal epithelial cells treated with CDEs under the CREPT-deletion condition. The result showed that CDEs dramatically altered the transcription profiles, with 2260 genes upregulated and 2300 genes down-regulated in MCF10A cells (Figure 6A), and 865 genes upregulated and 435 down-regulated in NMuMG cells (Figure S6A). KEGG analyses revealed that CDEs triggered the expression of multiple genes involved in several inflammation-related pathways, especially the TNF signaling pathway, in MCF10A (Figure 6B) and NMuMG (Figure S6B) cells. To verify the results from RNA-seq analyses, we performed RT-PCR experiments using the same batch of samples. The result showed that TNF, NOD2, CXCL1, and CSF1, downstream genes of the TNF signaling pathway, were upregulated in both MCF10A (Figure 6C) and NMuMG (Figure S6C) cells treated by CDEs. These results suggested that CDEs activated the TNF signaling pathway in the non-malignant epithelial cells, implying the role of inflammation in field cancerization.

To investigate the role of CREPT during the CDEs-induced field cancerization, we analyzed genes regulated by CREPT. The results showed that 2003 (out of 2260) genes in MCF10A cells and 477 (out of 865) genes in NMuMG cells failed to respond to the treatment of 231EXO or 4T1EXO when CREPT was deleted (Figure 6D and Figure S6D). We proposed that these genes are CDEs-induced genes that are also regulated by CREPT. Interestingly, these genes were also enriched in the TNF signaling pathway (Figure 5E and Figure S5E). We analyzed the expression levels of genes related to the TNF signaling pathway after CDEs treatment and found that the expression of TNFRSF1B (also known as TNFR2) and PIK3CD was regulated by CREPT in both groups (Figure 6F and Figure S6F). We verified that the mRNA levels of TNFRSF1B and PIK3CD were significantly increased in NMuMG and MCF10A cells treated with CDEs, but the elevation was significantly attenuated when CREPT was deleted (Figure 6G-H and Figure S6G-H). Furthermore, we found that phosphorylation levels of AKT and p65, downstream of TNFR2, were significantly increased in wild-type cells but not in CREPT-deletion cells after CDEs treatment (Figure 6I-K and Figure S6H). Taken together, these results suggest that CREPT is mainly involved in the regulation of CDEs-induced gene expression, in particular TNFR2 in the TNF signaling pathway, thereby affecting the transmission of TNF downstream signals.

## Discussion

**Figure 7:**
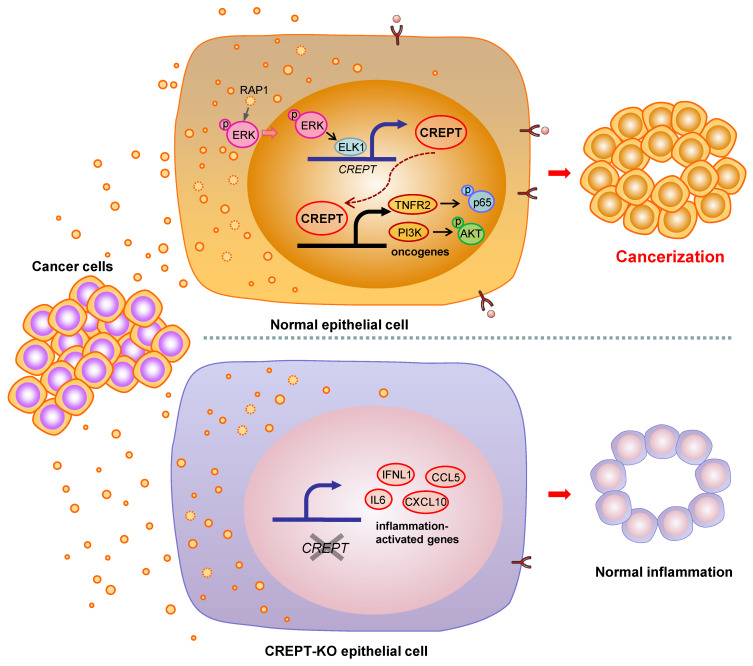
** Proposed model for field cancerization induced by CDEs.** CDEs are taken up by the adjacent normal epithelial cells, resulting in ERK activation and inflammatory responses. CREPT is elevated during CDEs-induction and further promotes oncogene expression, ultimately causing field cancerization. Without CREPT, the process of field cancerization is going to be interrupted.

Many studies have shown that the ostensibly normal tissues adjacent to the cancer are not completely normal 5, 9, 10. The adjacent tissues often have certain alterations both at the phenotype and gene expression profiles with a malignant tendency. This phenomenon, named field cancerization, occurs frequently approaching the primary tumor tissues due to the cancer insults or instigations 5, 11. Although the concept of field cancerization was first proposed in 1953, it has remained unclear which intrinsic factors response to the instigators. In this study, we verified the role of CDEs in field cancerization and suggested that CREPT is an intrinsic mediator to switch the CDEs-induced inflammatory responses into a malignant consequence. Our data supports a model in which CDEs activate ERK to induce CREPT expression, which enhances TNFR2 expression followed by the activation of survival signals (Figure 7). We propose that the induced CREPT expression switches the normal inflammation signaling into a malignant transformation signal and triggers field cancerization. Our study reveals a molecular mechanism for the occurrence of tumor-instigated filed cancerization, implying an important target for preventing the cancer local recurrence during the therapeutic practice.

Recently, extensive attention has been drawn to the roles of small extracellular vesicles in intercellular communications. The roles of small extracellular vesicles in the tumor microenvironment or distant metastatic organs are widely investigated, but their effect on the surrounding normal epithelial tissues is rarely reported. In 2020, Bisoffi group linked CDEs to field cancerization of paracancerous epithelial cells for the first time and defined EGR-1 and FASN as two field cancerization markers 17. In this study, we revealed that CDEs function as malignant insults in the tumor microenvironment by boosting inflammatory responses and activating a series of cancer-related gene expressions, which are further dependent on the expression of CREPT. Importantly, we demonstrated that field cancerization persists even under the CDEs withdrawal condition (see Figure 5). It appears that the alteration of normal epithelial cells caused by CDEs is sustainable, and the CDEs-triggered field cancerization could be a process on a one-way street, if CREPT expression is switched on.

The content of small extracellular vesicles is made up of multiple factors including proteins, metabolic substances, miRNAs, lncRNAs and even DNAs. Obviously, the components varied between NDEs and CDEs. Several reports identified key factors in CDEs for the promotion of malignancy. For instance, CDEs are reported to activate AKT via miR-21 28, or ILK protein in normal epithelial cells 18. Another study demonstrated that miR-183 was enriched in CDEs to promote the phosphorylation of p65 in macrophages 43. In this study, we explored the functional proteins differentially enriched in CDEs compared with NDEs. We have defined that CDEs contain proteins activating RAP1 signaling pathways. Furthermore, we verified that CDEs-induced ERK activation downstream of RAP1 is responsible for the CREPT expression. However, we could not exclude a possibility that miRNAs or other factors in CDEs might mediate CREPT expression. It remains to be of great interest to reveal how the proteins or other components in the CDEs alter the transcriptome of non-malignant epithelial cells.

Previous reports on small extracellular vesicles focused on the role of their internal substances, but few studies have explored the intrinsic factors of the recipient cells. In this study, we focused on endogenous proteins that play key roles in the recipient cells. We found that CREPT expression plays an important role in the non-malignant epithelial cells subject to field cancerization induced by CDEs. Our results showed that deletion of CREPT blocks field cancerization and over-expression of CREPT promotes cell proliferation. Moreover, CREPT has been reported as a candidate driver through high throughput analyses by Consortium group 38. We have observed an abundant expression of CREPT in several normal tissues adjacent to cancers, accompanied by hyperplasia or dysplasia (Figure 1). The expression level of CREPT exhibits a distance effect, with higher levels in normal tissues closer to the tumor, especially in the adjacent dysplasia tissues where CREPT expression is almost universally observed. The dysplasia tissues adjacent to cancer, or the tissues undergoing field cancerization, actually represent a state of precancerous lesions. Therefore, we propose that CREPT serves not only as a tumor marker but also as a marker for field cancerization or precancerous lesions. Clinically, since some tissues undergoing field cancerization may not exhibit morphological changes, it is hard for a pathologist or a surgery doctor to judge the indistinguishable cancerized boundary from completely normal tissues. Thus, the utilization of molecular markers such as CREPT for assessing surgical margins is highly valuable, as thorough removal of potentially cancerized tissues is crucial for preventing local recurrence.

Nevertheless, our results suggest that the CDEs-induced inflammation responses, especially TNFR2 signaling, trigger field cancerization dependent on the expression of CREPT. In another word, we propose that CREPT is an intrinsic mediator for field cancerization. TNFR2 is rarely expressed in normal cells but generally expressed on the surface of some regulatory T cells for activation of their proliferation. In addition, TNFR2 is also abundantly expressed on the surface of many human tumors 44. The expression of TNFR2 has been proven to promote cell survival through activation of AKT and NF-κB, which may be a key step in the inflammatory-cancer transition process. Our data indicate that CDEs induce the expression of TNFR2 in human and mouse epithelial cells and the induction of TNFR2 is regulated by CREPT. The control of TNFR2 expression by CREPT may be an important context for CDEs-induced field cancerization. Since CREPT is a co-activator for transcription, it is worth exploring which transcription factor coordinated with CREPT to promote the TNFR2 expression during field cancerization.

Inflammation is a double-edged sword in tumor formation. Many studies indicated that acute inflammation helps tumor cell clearance, but the chronic inflammation promotes tumorigenesis 14, 16, 45-48. In this study, we propose that CDEs induce the chronic inflammation for field cancerization. In our experiments, we exploited a relatively longer time to treat the cells with CDEs since short and pulse treatments with CDEs failed to promote any quick elevation of CREPT expression. On the other hand, a high dose and short pulse with CDEs could cause acute inflammation *in vitro*. In this context, the cells undergo apoptosis rather than field cancerization. We speculate that the effects of CDEs *in vivo* are chronic, as the secretion of small extracellular vesicles by cancer cells is a continuous process and the CDEs concentration during tumor progression is still limited. Our study echoes the observation of chronic inflammation during tumorigenesis. We believe that chronic inflammation, no matter whether caused by CDEs or other microenvironmental factors, is one of the driving forces to boost tumorigenesis. Importantly, our study supports the notion that the CREPT expression in the recipient cells determined the readout of the chronic inflammation. Interestingly, recently David Lyden et al. also reported that tumor extracellular vesicles induced secretion of TNF by Kupffer cells, generating a pro-inflammatory microenvironment 49, resulting in dysregulated metabolism and hepatic reprogramming. We believe that the CDEs-induced inflammatory response is most likely a systemic effect. As the CDEs-generated tumor insults always instigate non-malignant cells, inhibiting the expression of intrinsic factors such as CREPT will be a potential therapeutic strategy to block field cancerization.

## Conclusions

In conclusion, we suggest that CDEs lead to field cancerization of surrounding normal tissues, thereby promoting local recurrence of cancers. During this process, CREPT is elevated and functions as a gatekeeper. Up-regulation of CREPT could be used as a biomarker of field cancerization. Therefore, therapeutic targeting of CREPT may provide a viable strategy to disrupt early pro-tumorigenic alterations, and to prevent local recurrence of cancers.

## Material and Methods

### Plasmids, antibodies and inhibitors

The plasmids (pcDNA3.1/ Myc-CREPT and pcDNA3.1/ flag-ELK1) and the CRISPR/Cas9-mediated CREPT deletion plasmid were constructed in our lab 33. Antibodies against Alix (#2171), ERK1/2 (#4695), p-AKT (Ser473, #9271), AKT (#9272), p-p38 (Thr180/Tyr182, #4511), p38 (#8690), p-p65 (Ser536, #3033) and p65 (#8242) were purchased from Cell Signaling Technology, USA. The anti-p-ERK1/2 antibody (sc-7383) and anti-Calnexin antibody (sc-23954) were purchased from Santa Cruz Biotechnology. The anti-Flotillin 1 antibody (15571-1-AP) was purchased from Proteintech, China. Antibodies against CD9 (MA5-31980), CD63 (PA5-92370) and CD81 (MA5-32333) were purchased from ThermoFisher, USA. The anti-Actin antibody (A5316) was from Sigma. An anti-CREPT antibody (3E10) was raised in our lab. Small extracellular vesicle secretion inhibitor, GW4869, was purchased from Sigma. ERK inhibitor, SCH772984, was purchased from Shanghai Yuanye Bio-Technology, China. JNK inhibitor, SP600125, was purchased from Abcam, USA. p38 inhibitor (SB203580) was purchased from Beyotime, China.

### Immunohistochemical (IHC) staining

All human cancer tissues were collected from Ensinger lab at the Medical University of Innsbruck and Morii lab at Osaka University. The slides of the mouse skin tissues adjacent to the PDX tumor and the opposite normal skin tissues were from Prof. Liu Suling's lab at Fudan University, China. The patient information is provided in Table S1. Tissues were kept and stained according to the routine protocol 50.

### Co-culture system

Co-culture inserts (0.4-μm pores; Corning, USA) were placed into 6-well culture plates. NMuMG cells were added to the lower chambers. To determine the role of small extracellular vesicles, NMuMG and 4T1 cells were pre-treated by DMSO or GW4869 (5 µM) for 4 days, then the treated NMuMG and 4T1 cells with the addition of DMSO or GW4869 (in the upper chambers) were co-cultured with NMuMG cells (in the lower chambers) for 4 days. At the end of the experiments, NMuMG cells in the lower chambers were harvested to detect CREPT expression through Western blotting.

### Cell culture and transfection

Mouse breast cancer cell line 4T1 (RRID: CVCL_0125), and mouse non-malignant breast epithelial cell line NMuMG, were kindly provided by Prof. Zheng Hanqiu. Human breast cancer cell line MDA-MB-231 and human non-malignant breast epithelial cell line MCF10A (RRID: CVCL_0598) were from ATCC. Human colorectal cancer cell line SW620 was kindly provided by Prof. Wang Dong, and human immortalized colonic epithelial cell line NCM460 (RRID: CVCL_0460) was kindly provided by Prof. Wu Wei. Mouse melanoma cell line B16 (RRID: CVCL_F936) and Chinese hamster ovary cell line CHO (RRID: CVCL_0213) were from our lab. SW620 was cultured in L-15 medium supplemented with 10% fetal bovine serum (FBS). NCM460 and B16 were maintained in RPMI-1640 medium supplemented with 10% FBS. 4T1, NMuMG and MDA-MB-231 were grown in DMEM supplemented with 10% FBS. MCF10A was cultured in 5% horse serum-DMEM/F12 medium with 10 µg/mL insulin, 0.1 µg/mL cholera toxin, 0.5 µg/mL hydrocortisone and 20 ng/mL epidermal growth factor (EGF). All cell lines were maintained at 37°C with 5% CO_2_, except for SW620, which was cultured in an incubator containing atmospheric air at 37°C. Cells were transfected with plasmids using Vigofect (Vigorous Inc., China), according to the manufacturer's instruction. To generate stable CREPT over-expression cell lines, CHO, NMuMG and MCF10A were transfected by pcDNA3.1/Myc-CREPT and screened by neomycin. For CREPT deletion cell lines, CHO, NMuMG and MCF10A were transfected by PX458 vector or PX458 carrying sgRNAs against CREPT and screened by green fluorescence through flow cytometry. The clones were randomly picked up and identified by Western blotting.

### Isolation of small extracellular vesicles

Cells were seeded in 150 mm × 25 mm Corning culture dishes in their respective medium. When cells were grown until about 80% confluency, complete medium was replaced with 10% vesicle-depleted FBS supplemented medium. FBS was centrifuged at 110 000 × g for 16 h to remove the small extracellular vesicles. After 24~48 h following incubation, conditioned medium (CM) was collected and pooled (160 mL total), and centrifuged at 300 × g for 5 min followed by 2000 × g for 10 min, to remove cell debris and apoptotic vesicles. CM was then passed through a 0.22 µm filter to remove microvesicles, followed by ultracentrifugation at 110 000 × g for 1.5 h at 4°C. The remaining pellet was re-suspended in PBS and washed (110 000 × g for 1.5 h at 4°C), and the pellet was reconstituted in PBS, aliquoted, and stored at -80°C.

### Transmission Electron Microscopy

For negative staining, a drop of small extracellular vesicles was incubated for 2 min on a copper grid covered with formvar film, stabilized by carbon. Then, the excess liquid was removed by filter paper. The grid was next put on a drop of distilled water and removed the excess water by filter paper immediately. After that, the grid was exposed for 2 min to a drop of 2% uranyl acetate and removed the excess liquid by filter paper. The grids holding the adsorbed small extracellular vesicles were examined under a transmission electron microscope (TEM) (JEM 1400, Jeol, Tokyo, Japan, with a digital camera Veleta, EMSIS, Münster, Germany). Small extracellular vesicle measurements were made directly on the camera screen using iTEM (EMSIS, Münster, Germany) software.

### Nanoparticle Tracking Analysis

Small extracellular vesicle quantities and particle concentrations were analyzed using a nanoparticle tracking analysis (NTA) system, NanoSight LM14 (Malvern, Surrey, UK). Depending on the concentration of the particles, the samples were diluted 100-fold in PBS to obtain optimal conditions for NTA concentration measurements. Each sample was measured in triplicate, with a camera setting of 16, an acquisition time of 30 s, and a detection threshold setting of 5. At least 200 completed tracks were analyzed per video. NTA analytical software version 3.1 was used for data analysis and capture.

### Western Blotting

Experiments were performed according to protocols in the lab 51. Briefly, cell lysates were prepared by treating cells with lysis buffer. Proteins were denatured by heating at 100°C for 10 minutes, separated on a 10% sodium dodecyl sulfate-polyacrylamide gel electrophoresis (SDS-PAGE) gel, and transferred to a nitrocellulose membrane. After transfer, the membrane was blocked in 10% non-fat milk or bovine serum albumin (BSA) blocking buffer for 1 h at room temperature to minimize non-specific binding. Next, the membrane was incubated with the primary antibody diluted in blocking buffer (1:1000) overnight at 4°C. Subsequently, the membrane was incubated with a secondary antibody for 1 h at room temperature. Western blotting results were visualized with MiniChemi610 Imaging System (Sagecreation Service For Life Science, China). Densitometric analysis was performed using ImageJ software to quantify protein expression levels. All experiments involving Western blotting were performed in triplicate, and representative blot images were presented.

### Luciferase activity analysis

CHO was transfected with the recombination plasmid of pcDNA3.1 (RRID: Addgene_79663) and pGL3-CREPT-promoter and screened by neomycin to get a stable cell line. Then, the cells were treated with CDEs in different concentrations for 3 days. The luciferase reporter activity was examined using the Dual-Lucy Assay Kit (Vigorous, China) and read by the microplate reader (Enspire, PerkinElmer, USA).

### Cell proliferation Assay

Cell proliferative ability was evaluated by Cell Count Kit-8 (CCK-8) assay or colony formation assay. For CCK-8 assay, cells were plated onto a 96-well plate (Corning), at a density of 1 × 10^3^ cells/well and cultured for the indicated time. 10 µL of CCK-8 reagent (Dojindo Molecular Technologies, Japan) and 100 μL of medium were added to the plates, which were then incubated with 5% CO_2_ at 37°C for 2 h. After the incubation, the optical density (OD450) value was assessed by a microplate reader (BioTek, USA) for the evaluation of cell proliferation ability. For colony formation assay, cells were plated onto a 6-well plate (Corning, USA), at a density of 500 cells/well. After 1~ 2 weeks, the medium was removed and cells were washed with PBS twice. Then, the cells were staining with 0.1% crystal violet for 30 min. The plates were scanned and the colonies were analyzed by Image J (RRID:SCR_003070).

### Sample preparation, Tandem Mass Tag (TMT)-labeling, LC-MS/MS analysis and protein identification and quantification

The quantitative proteomics analysis was performed according to a routine protocol 52. For TMT-labeling proteomic analysis, small extracellular vesicles from SW620 and NCM460 cells were extracted three times independently for trypsin digestion. Then, TMT 6-plex reagent (90061; Thermo Scientific) was used to label SW620EXO and NCM460EXO. Control NCM460-EXO samples C1, C2, C3 were tagged with TMTs 126.1, 127.1 and 128.1 and SW620-EXO samples A1, A2, A3 with TMTs 129.1, 130.1 and 131.1, respectively, for the next LC-MS/MS analysis. Protein identifications, quantifications, and database searches were performed by Proteome Discoverer (RRID:SCR_014477). The raw files from the fractions were searched against a *Homo sapiens* Swiss-Prot UniProt protein database (www.uniprot.org/). The protein ratio was computed as 129+130+131 over 126+127+128. Differentially expressed proteins were selected with protein ratio > 1.5 or < 0.67 above the 95% confidence level in the comparison. Channel 126 was used for labeling the internal reference sample. The raw data and the analysis result are presented in Supplementary Table 2.

### Bioinformatics analysis

The differentially expressed proteins from TMT-labeling proteomics or the identified proteins from label-free proteomics were carried out by bioinformatics analysis. The KEGG analyses were performed by KOBAS 3.0 53. The volcano plot, heatmap, and bubble diagram of KEGG analyses were performed using the OmicShare tools, an online platform for data analysis (http://www.omicshare.com/tools).

### Real-time PCR analysis

Total RNA was extracted using TRIzol (Invitrogen, USA). RNA was reverse transcribed using a Quantscript RT Kit (TIANGEN Biotech, China). RT-qPCR was performed using a Talent qPCR PreMix (SYBR Green) Kit (TIANGEN Biotech, China) on a Bio-Rad machine using the following conditions: pre-denaturation at 95°C for 3 min; denaturation at 95°C for 5 s, annealing and extension at 60°C for 15 s, 40 cycles. Gene expression was measured using the 2^-ΔΔCt^ formula and the mRNA level of Actin as the control.

### qPCR Primers Sequence

Mouse Cxcl1_F TGGCTGGGATTCACCTCAAG

Mouse Cxcl1_R CCGTTACTTGGGGACACCTT

Mouse Csf1_F CTTCAGCCACTAGCGAGCAA

Mouse Csf1_R CCCAGCCATGTCGAAGAAGG

Mouse Nod2_F AACTAGCTCTCTTCAACAACAAACT

Mouse Nod2_R TGATTGTTCCCCACCCTCAG

Mouse Tnf_F CAGCCGATGGGTTGTACCTT

Mouse Tnf_R GTGTGGGTGAGGAGCACGTA

Mouse Tnfrsf1b_F GGCTGTCTCCCACTTGTAGC

Mouse Tnfrsf1b_R CGAGATGACAGAACCCGTCT

Mouse Pik3cd_F GCCCCAAACCAAGGAGATGA

Mouse Pik3cd_R GCTCCACACAGACTTCCTCC

Mouse Crept_F GTCTGTGCTTGTGGATGCTT

Mouse Crept_R GTATGAACTCGCCGCCGTA

Mouse Actin_F GTGACGTTGACATCCGTAAAGA

Mouse Actin_R GCCGGACTCATCGTACTCC

Human CXCL1_F CTGGCTTAGAACAAAGGGGCT

Human CXCL1_R TAAAGGTAGCCCTTGTTTCCCC

Human CSF1_F GAAAGTTTGCCTGGGTCCTCT

Human CSF1_R AGGAGACAGACCAACAACAGC

Human NOD2_F AGTGGGGTTTTTCAGTGAGGG

Human NOD2_R CTGTCTACCAACCCCACCTTC

Human TNF_F TGGGATCATTGCCCTGTGAG

Human TNF_R GGTGTCTGAAGGAGGGGGTA

Human TNFRSF1B_F GCATTTACACCCTACGCCCC

Human TNFRSF1B_R CTCACAGGAGTCACACACGG

Human PIK3CD_F ATGTCACCGAGGAGGAGCA

Human PIK3CD_R AGTGCTCCTGGACTTCATGC

Human CREPT_F TGTCCCTTTGGCTCATCCAC

Human CREPT_R CATCTGCCTCTCTGGCAACA

Human ACTIN_F CATGTACGTTGCTATCCAGGC

Human ACTIN_R CTCCTTAATGTCACGCACGAT

### Nude mouse tumor formation assay

A total of 5 × 10^6^ NMuMG CREPT-WT/KO cells treated or untreated with 4T1EXO were orthotopically injected into the mammary pads of athymic nude mice which were euthanized 10 weeks post-injection. A total of 5 × 10^6^ CHO cells with/without CREPT over-expression were injected subcutaneously into the two flanks of the nude mice which were euthanized 5 weeks post-injection. At the end of the experiments, mice were sacrificed by the euthanasia method of carbon dioxide inhalation. The mass of the tumors was measured by an analytical balance.

### Mouse strains and treatments

To generate mammary-specific CREPT knockout mice, we crossed BALB/c; *MMTV-Cre* mice with mice homozygous for a floxed allele of CREPT (*CREPT^fl/fl^*). *MMTV-Cre^+/-^CREPT^fl/fl^* mice were then maintained by breeding to *CREPT^fl/fl^* mice. In *MMTV-Cre^-/-^CREPT^fl/fl^* (wild-type) control mice, we injected 100 μl of PBS, NMUEXO (1×10^9^ particles) or 4T1EXO (1×10^9^ particles) at the same location in the mammary fat pad once a day for 14 consecutive days. In *MMTV-Cre^+/-^CREPT^fl/fl^* (CREPT-knockout) mice, we injected 100 μl of 4T1EXO (1×10^9^ particles) at the same location in the mammary fat pad once a day for 14 consecutive days. At the end of the experiment, the mice were euthanized, and the mammary fat pad tissue at the target site was clipped for fixation.

To generate inducible systemic CREPT knockout mice, we crossed B6; *ERT2(a G400V/M543A/L544A triple mutation of the human estrogen receptor)-Cre* 54, 55 mice expressing 4-OH-tamoxifen-sensitive Cre with mice homozygous for a floxed allele of CREPT (*CREPT^fl/fl^*). *ERT2-Cre^+/-^CREPT^fl/fl^* mice were then maintained by breeding to *CREPT^fl/fl^* mice. *ERT2-Cre^+/-^CREPT^fl/fl^* mice were treated with intraperitoneal injections of 20 μg of Tamoxifen (Sigma) per gram of mouse weight (diluted in 100 μl of sunflower seed oil with 10% ethanol) or an equal volume of oil once a day for five consecutive days, then wait for a week to get CREPT-knockout mice or wild-type mice. In wild-type mice, we injected 100 μl of PBS, CHOEXO (1×10^9^ particles), or B16EXO (1×10^9^ particles) at the same location in the skin once a day for 14 consecutive days. In CREPT-knockout mice, we injected 100 μl of B16EXO (1×10^9^ particles) at the same location in the skin once a day for 14 consecutive days. At the end of the experiment, the mice were euthanized, and the skin tissue at the target site was clipped for fixation.

### RNA Sequencing

#### CHO KO/WT/OE

Total RNAs were extracted from CHO CREPT-WT, CREPT-KO and CREPT-OE cells using TRIzol (Invitrogen) according to manufacturers' recommendations. High-throughput sequencing was performed by Illumina Hiseq platform (Novogene). The RNA-seq results were mapped to the CriGri_1.0 genome sequence from NCBI. The clean reads were mapped to reference transcripts using TopHat, and then calculate the gene expression level for each sample with HTSeq v0.6.1. DESeq R package (1.20.0) were used to identify the differentially expression genes. The results of FPKM values and the differential expression analysis are presented in Supplementary Table 3. The P values were adjusted using the Benjamini & Hochberg method. Corrected P-value of 0.005 and log2 (Fold change) of 1 were set as the threshold for significantly differential expression, then used KOBAS software to test the statistical enrichment of differential expression genes in KEGG pathways.

#### NMuMG WT/KO+/-4T1EXO&MCF10A WT/KO+/-231EXO

Total RNAs were extracted from NMuMG CREPT-WT and CREPT-KO cells treated with/without 4T1EXO for 2 weeks and MCF10A CREPT-WT and CREPT-KO cells treated with/without 231EXO for 2 weeks using TRIzol (Invitrogen). High-throughput sequencing was performed by Illumina Novaseq 6000 platform (Shanghai Majorbio Bio-pharm Technology). The RNA-seq results were mapped to the GRCm39 or GRCh38.p13 genome sequence from NCBI. The clean reads were mapped to reference transcripts using HISAT2, and then calculated the gene expression level for each sample with RSEM. DEGseq algorithms were used to identify the differential expression genes. Genes with p-adjust < 0.001 and |Fold change| >= 2 were used to analyze. The data were analyzed on the free online platform of Majorbio Cloud Platform (www.majorbio.com). The RNA-seq raw data was submitted to NCBI GEO under accession number GSE249383.

### Statistical Analysis

Statistical calculations were performed using GraphPad Prism 8 software (La Jolla, CA, USA, RRID:SCR_002798). All experiments were performed at least three times. Data were expressed as means with standard errors. To evaluate differences, unpaired and two-tailed t-tests were performed to compare means for two groups and two-way ANOVA tests were performed to determine how a response is affected by two factors. Asterisks indicate a significant difference (*p < 0.05, **p < 0.01, ***p < 0.001, ****p < 0.0001). NS stands for no significant differences.

## Supplementary Material

Supplementary figures and table 1.

Supplementary table 2.

Supplementary table 3.

## Figures and Tables

**Table 1:** Enhanced Expression of CREPT in Tumors Adjacent Tissues

	Expression^a^ %			
Position	Strong	Median	Week	Negative
Primary tumor	66.7 (22/33)	27.3 (9/33)	0 (0/33)	6.1 (2/33)
Dysplasia	52.0 (13/25)	36.0 (9/25)	12.0 (3/25)	0 (0/25)
Normal(proximal)	33.3 (7/21)	38.1 (8/21)	28.6 (6/21)	0 (0/21)
Normal(distal)	8.3 (1/12)	33.3 (4/12)	25.0 (3/12)	33.3 (4/12)

^a^ The intensity of CREPT expression was assessed by immunohistochemistry. The number in parentheses represents the ratio of the number of cases at this intensity level in the CREPT expression to the total number of cases.

## Abbreviations

AKT: Protein Kinase B
CREPT: Cell-cycle Related and Expression-elevated Protein in Tumor
CDEs: cancer-derived small extracellular vesicles
ERK: Extracellular Regulated protein Kinases
EXO: small extracellular vesicles
IHC: Immunohistochemistry
JNK: c-Jun N-terminal kinase
MAPK: Mitogen-activated protein kinases
NF-kB: Nuclear factor kappa-B
PCR: Polymerase chain reaction
PI3K: Phosphoinositide 3-Kinase
PDX: Patient-Derived Xenograft
TNF: Tumor Necrosis Factor
TNFR2: TNF receptor 2
