# Supplementary material for: Microenvironment-induced CREPT expression by cancer-derived small extracellular vesicles primes field cancerization
Source: Theranostics. 2024 Jan 1;14(2):662–80. doi: 10.7150/thno.87344 (PMC10758052; doi:10.7150/thno.87344)
Supplement: Supplementary file 1 — Supplementary figures and table 1. [file thnov14p0662s1.pdf]

Supplementary materials

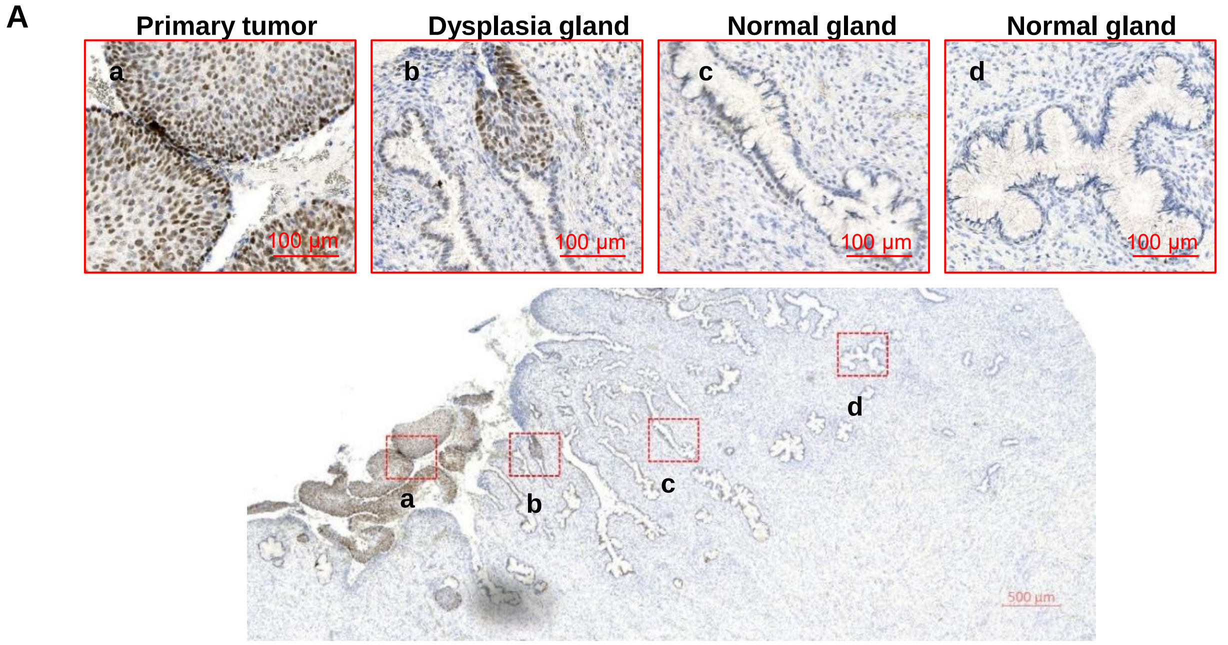

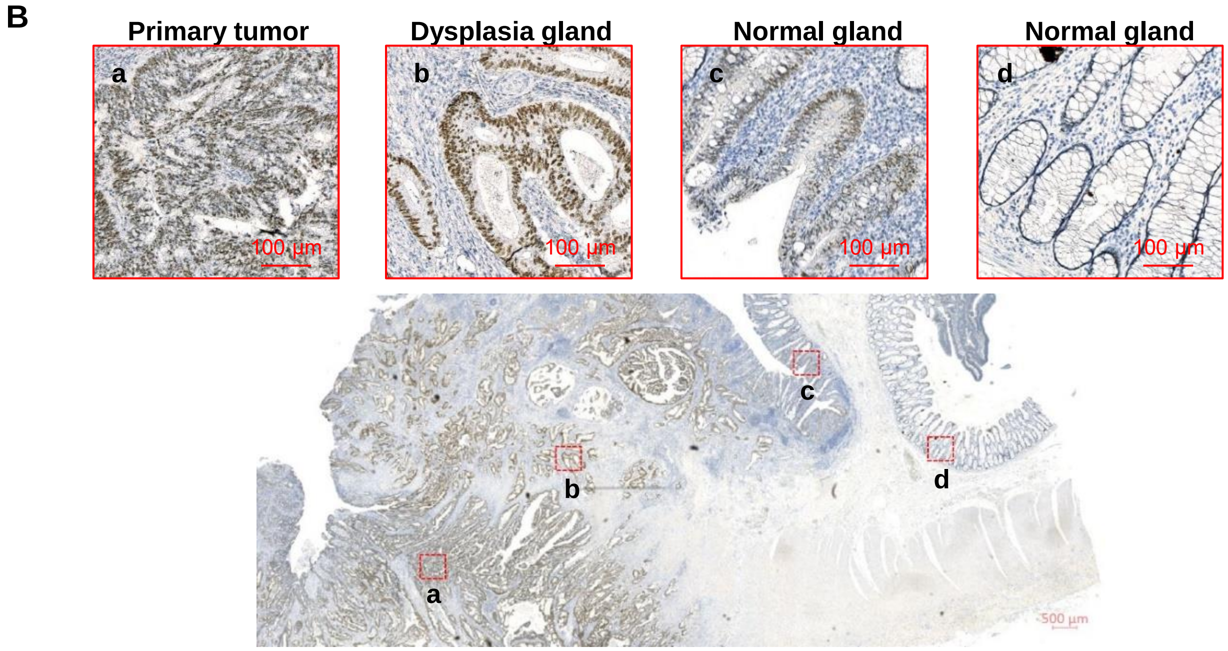

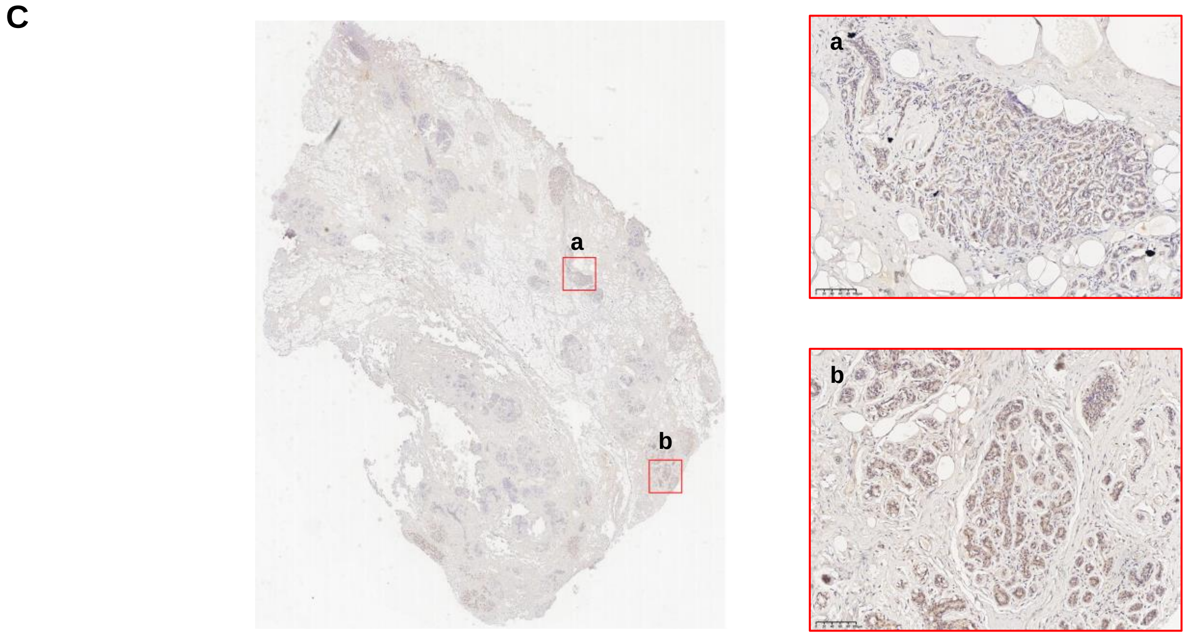

Figure S1, Related to Figure 1. CREPT is upregulated in the cancerized field adjacent to the tumor. IHC staining of CREPT in human cervical carcinoma (A) and colorectal carcinoma (B). CREPT is highly expressed in the primary tumor (a), dysplasia (b) and the proximal normal glands (c), but not expressed in the distal normal glands (d). (C) IHC staining of CREPT in human hyperplastic mammary tissue.

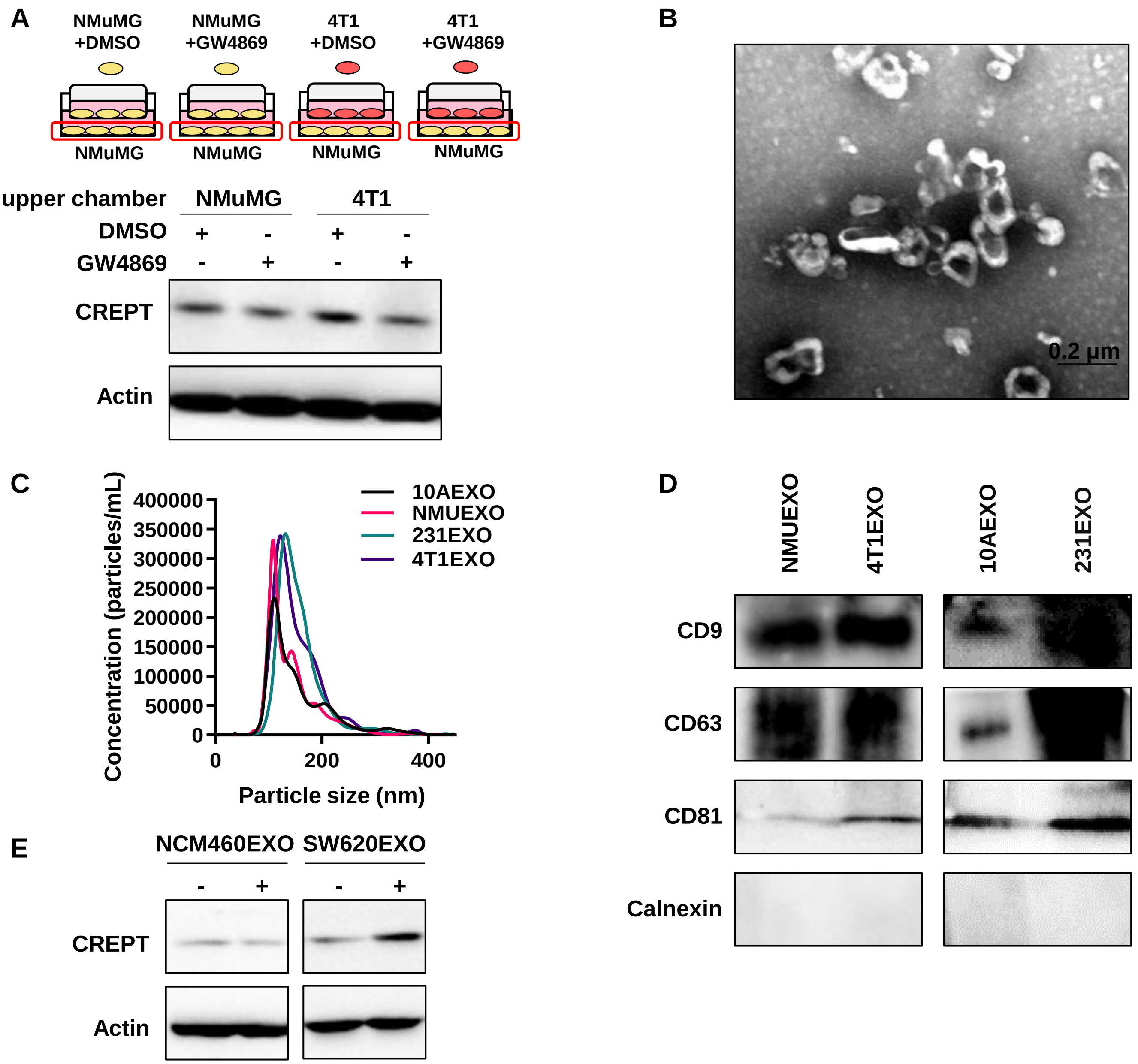

Figure S2, Related to Figure 2. CDEs induce CREPT expression in non-malignant epithelial cells. (A) Western blot of CREPT in protein extracts of NMuMG cells (in the lower chamber) cultured with NMuMG/ 4T1 cells (in the upper chamber) treated with DMSO/ GW4869 (5 μM). (B) Representative TEM image of small extracellular vesicles from MDA-MB-231 cells confirmed the expected teacup-shaped morphology of vesicles. (C) Nanoparticle Tracking Analysis of small extracellular vesicles derived from MDA-MB-231, 4T1, MCF10A and NMuMG cells showed the size distributed between 30-200 nm. (D) Western blot of CD9, CD63, CD81 and Calnexin in protein extracts of small extracellular vesicles from NMuMG, MCF10A, 4T1 and MDA-MB-231 cells. (E) Western blot of CREPT in protein extracts of NCM460 cells treated for 3 days with/ without NCM460EXO or SW620EXO in the concentration of 1×10^9 particles/mL.

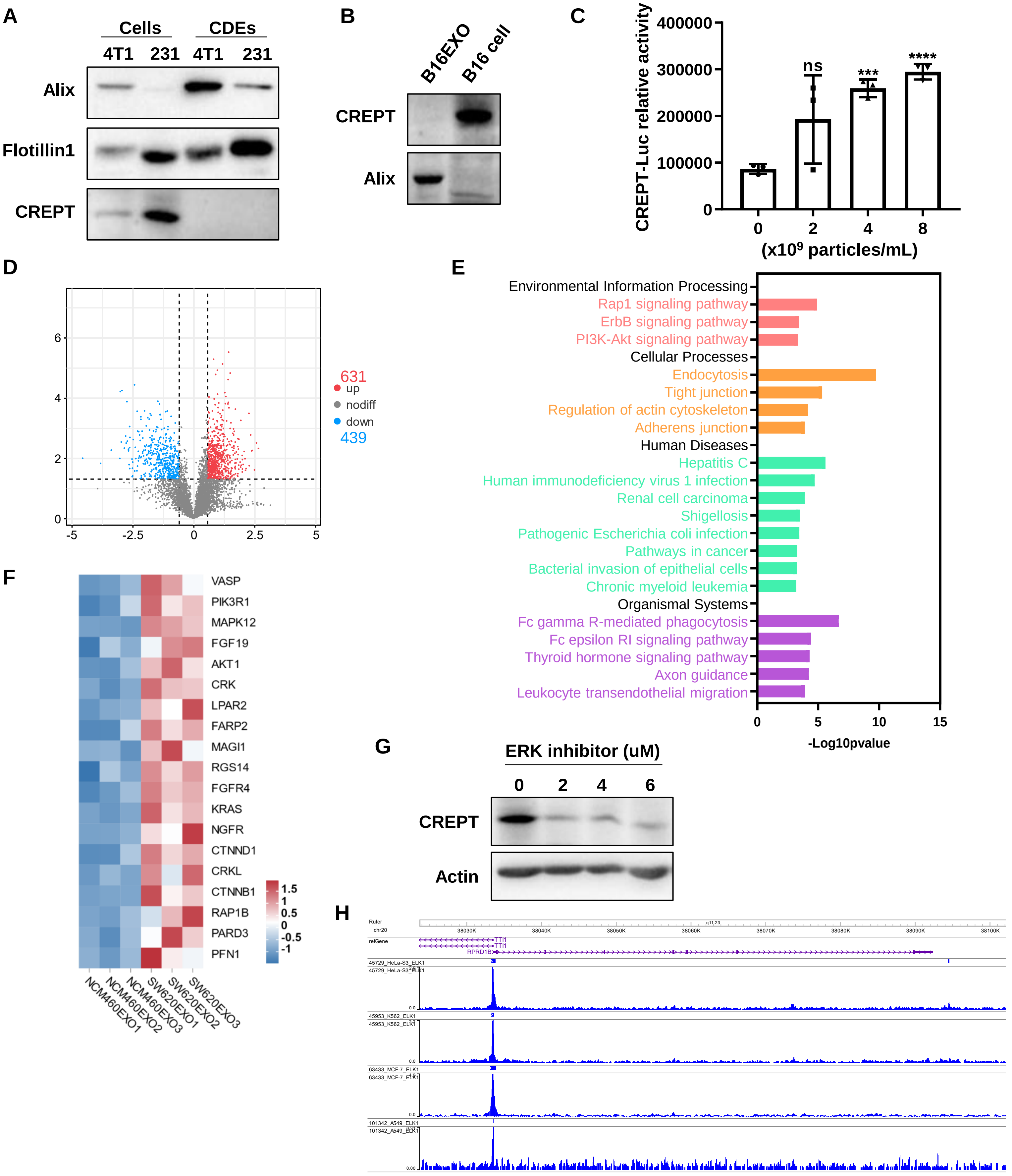

A
Cells
CDEs
4T1
231
4T1
231
Alix
Flotillin1
CREPT
B
B16EXO
B16 cell
CREPT
Alix
C
CREPT-Luc relative activity
400000
300000
200000
100000
0
ns
***
****
0
2
4
8
(x10^9 particles/mL)
D
631
up
nodiff
down
439
-5
-2.5
0
2.5
5
E
Environmental Information Processing
Rap1 signaling pathway
ErbB signaling pathway
PI3K-Akt signaling pathway
Cellular Processes
Endocytosis
Tight junction
Regulation of actin cytoskeleton
Adherens junction
Human Diseases
Hepatitis C
Human immunodeficiency virus 1 infection
Renal cell carcinoma
Shigellosis
Pathogenic Escherichia coli infection
Pathways in cancer
Bacterial invasion of epithelial cells
Chronic myeloid leukemia
Organismal Systems
Fc gamma R-mediated phagocytosis
Fc epsilon RI signaling pathway
Thyroid hormone signaling pathway
Axon guidance
Leukocyte transendothelial migration
0
5
10
15
-Log10pvalue
F
VASP
PIK3R1
MAPK12
FGF19
AKT1
CRK
LPAR2
FARP2
MAGI1
RGS14
FGFR4
KRAS
NGFR
CTNND1
CRKL
CTNNB1
RAP1B
PARD3
PFN1
1.5
1
0.5
0
-0.5
-1
NCM460EXO1
NCM460EXO2
NCM460EXO3
SW620EXO1
SW620EXO2
SW620EXO3
G
ERK inhibitor (uM)
0
2
4
6
CREPT
Actin
H
Ruler
chr20
refGene
RPRD1B
45729_HeLa-S3_ELK1
45953_K562_ELK1
63433_MCF-7_ELK1
101342_A549_ELK1

Figure S3, Related to Figure 3. CDEs induce CREPT expression through ERK activation. (A) Western blot of Alix, Flotillin 1 (two common exosomal markers) and CREPT in protein extracts of cells and small extracellular vesicles from 4T1 and MDA-MB-231 cells. (B) Western blot of Alix and CREPT in protein extracts of cells and small extracellular vesicles from B16 cells. (C) The luciferase reporter activity in CHO cells transfected with a luciferase gene driven by CREPT-promoter and treated for 24 h with 0, 2, 4 and 8 $\times$ 10^9 particles/mL B16EXO. (D) The volcano plot of differentially enriched proteins in SW620EXO compared with NCM460EXO. Red dots indicate 631enriched proteins (fold change > 1.5, p-value < 0.05), blue dots indicate 439 reduced proteins (fold change < 0.67, p-value < 0.05). (E) KEGG analysis of the 631 differentially enriched proteins in SW620EXO compared with NCM460EXO. The -Log10(p-value) is indicated. (F) The heatmap of relative levels of 19 proteins associated with RAP1 signaling pathway enriched in SW620EXO. (G) Western blot of CREPT in protein extracts of 4T1 cells treated with ERK inhibitor (SCH772984) in the concentration of 0, 2, 4 and 6 µM. (H) The ChIP-Seq results from Cistrome DB showed ELK1 occupied the CREPT promoter region of Hela-S3, K562, MCF7 and A549 cells.

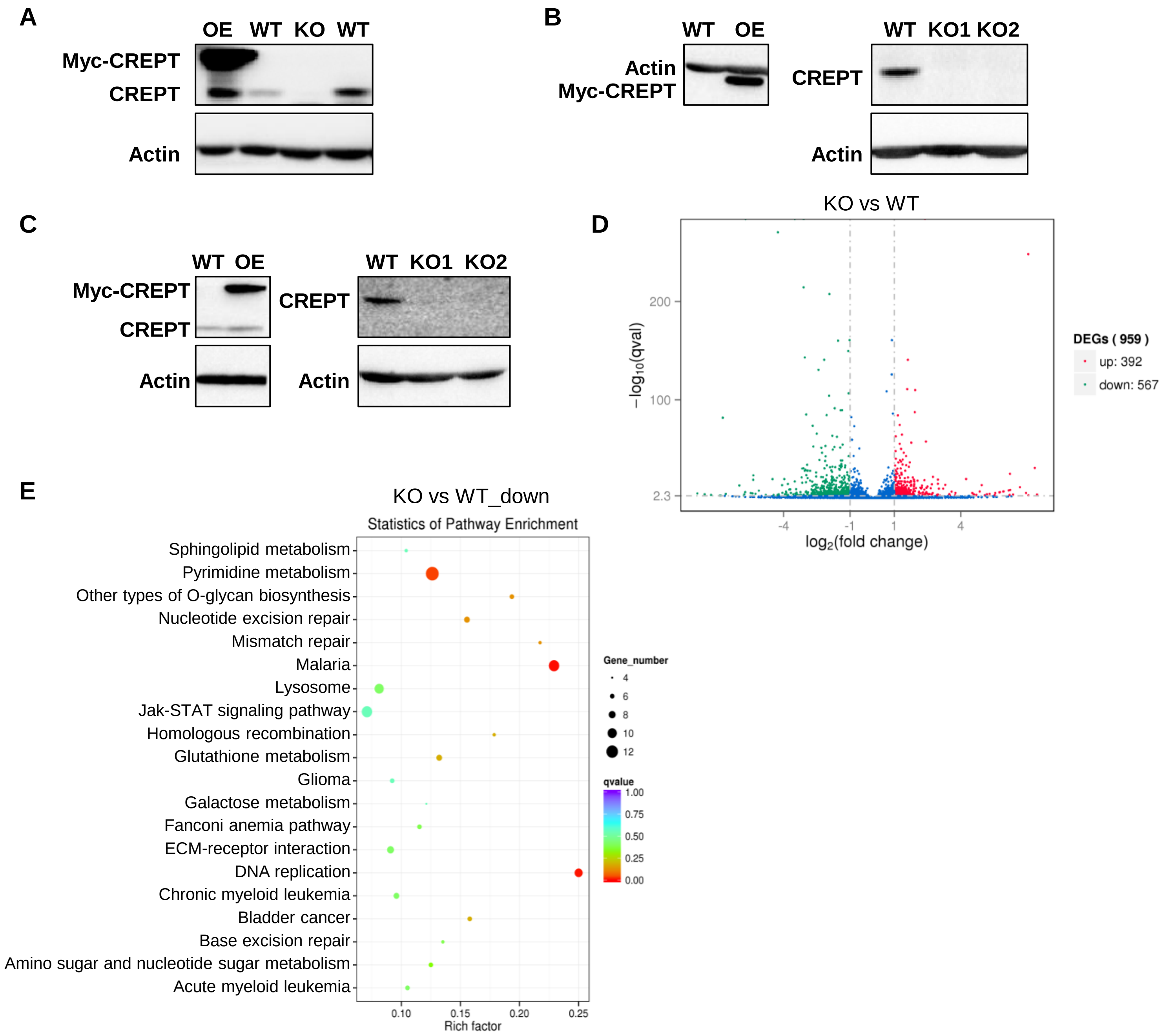

Figure S4, Related to Figure 4. Over-expression of CREPT enhances the proliferation of non-malignant epithelial cells. Western blot results showing CREPT over-expression (OE) and knockout (KO) in CHO (A), NMuMG (B), and MCF10A (C) cell lines. Actin was used as a control. WT, wild-type. (D) The volcano plot of DEGs after CREPT knockout in CHO. (E) The bubble plot of KEGG enrichment analysis of down-regulated genes after CREPT knockout in CHO.

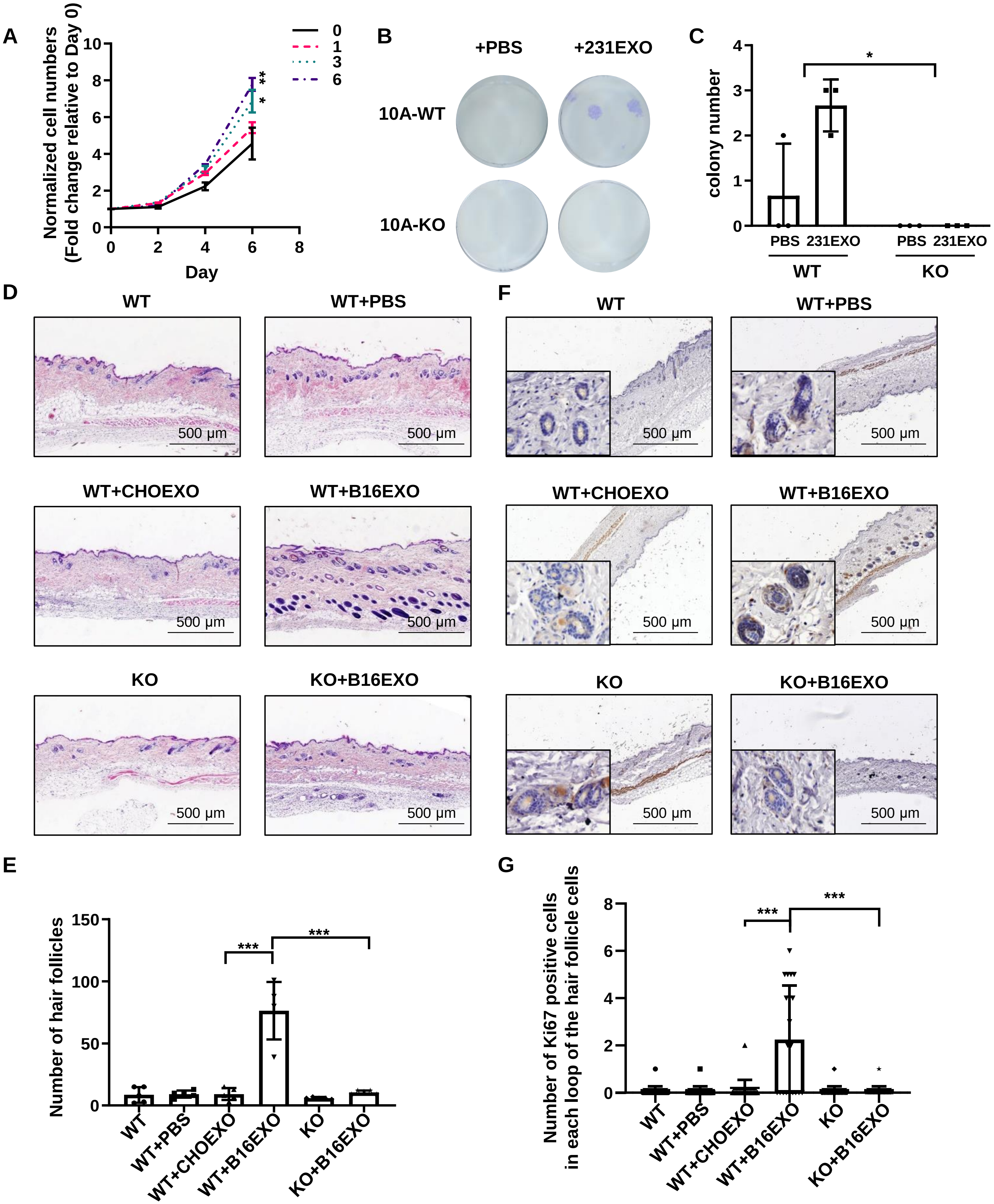

A
B
C
D
E
F
G
Normalized cell numbers (Fold change relative to Day 0)
Day
0
1
3
6
**
*
+PBS
+231EXO
10A-WT
10A-KO
colony number
PBS
231EXO
WT
KO
*
WT
WT+PBS
WT+CHOEXO
WT+B16EXO
KO
KO+B16EXO
500 μm
Number of hair follicles
***
Number of Ki67 positive cells in each loop of the hair follicle cells

Figure S5, Related to Figure 5. CREPT is required for cancerization induced by CDEs. (A) CCK-8 assay during 6 days of NMuMG cells treated with 4T1EXO for 0, 1, 3 or 6 days. The cell number indicated by OD450 is normalized by the value of day 0. (B) Representative images of cells stained with crystal violet showing colony formation from MCF10A CREPT-WT and CREPT-KO treated with PBS or 231EXO for two weeks. (C) Quantification of colony formation assay (B), the data shows the colony number of each group. (D) The HE staining of the skin in wild-type mice (n=5) after the injection of PBS, CHOEXO or B16EXO in the skin for 14 days and the skin in the mice with systemic knockout of CREPT (n = 5) after the injection of B16EXO in the skin for 14 days. (E) The statistical results of Figure S5D, n = 5. (F) IHC staining of Ki67 in the corresponding skin tissue in Figure S5D. (G) The statistical results of Figure S5F, n = 20.

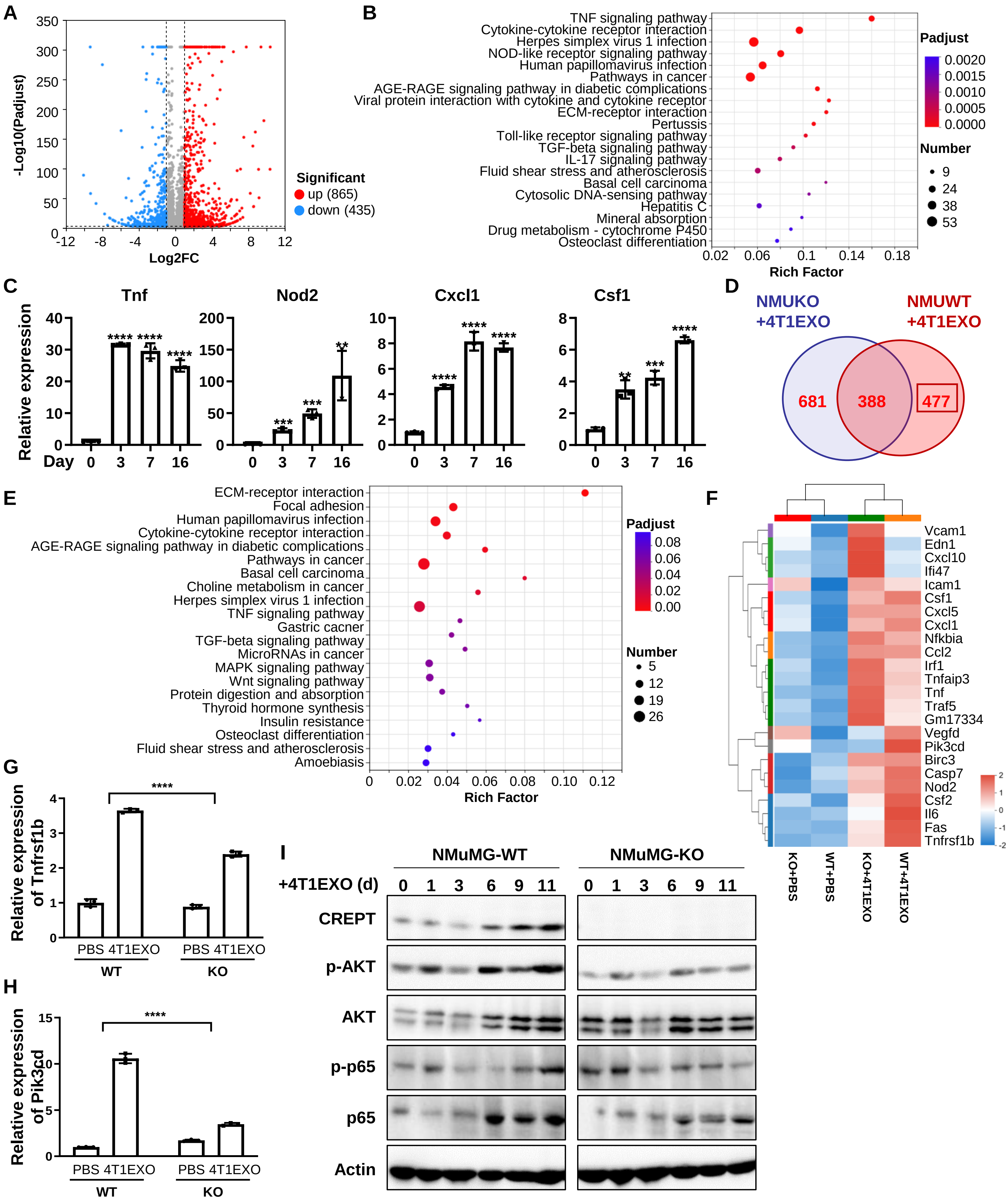

A
-Log10(Padjust)
Log2FC
Significant
up (865)
down (435)
B
TNF signaling pathway
Cytokine-cytokine receptor interaction
Herpes simplex virus 1 infection
NOD-like receptor signaling pathway
Human papillomavirus infection
Pathways in cancer
AGE-RAGE signaling pathway in diabetic complications
Viral protein interaction with cytokine and cytokine receptor
ECM-receptor interaction
Pertussis
Toll-like receptor signaling pathway
TGF-beta signaling pathway
IL-17 signaling pathway
Fluid shear stress and atherosclerosis
Basal cell carcinoma
Cytosolic DNA-sensing pathway
Hepatitis C
Mineral absorption
Drug metabolism - cytochrome P450
Osteoclast differentiation
Rich Factor
Padjust
Number
C
Tnf
Nod2
Cxcl1
Csf1
Relative expression
Day
D
NMUKO +4T1EXO
NMUWT +4T1EXO
681
388
477
E
ECM-receptor interaction
Focal adhesion
Human papillomavirus infection
Cytokine-cytokine receptor interaction
AGE-RAGE signaling pathway in diabetic complications
Pathways in cancer
Basal cell carcinoma
Choline metabolism in cancer
Herpes simplex virus 1 infection
TNF signaling pathway
Gastric cacner
TGF-beta signaling pathway
MicroRNAs in cancer
MAPK signaling pathway
Wnt signaling pathway
Protein digestion and absorption
Thyroid hormone synthesis
Insulin resistance
Osteoclast differentiation
Fluid shear stress and atherosclerosis
Amoebiasis
Rich Factor
Padjust
Number
F
Vcam1
Edn1
Cxcl10
Ifi47
Icam1
Csf1
Cxcl5
Cxcl1
Nfkbia
Ccl2
Irf1
Tnfaip3
Tnf
Traf5
Gm17334
Vegfd
Pik3cd
Birc3
Casp7
Nod2
Csf2
Il6
Fas
Tnfrsf1b
KO+PBS
WT+PBS
KO+4T1EXO
WT+4T1EXO
G
Relative expression of Tnfrsf1b
PBS 4T1EXO
PBS 4T1EXO
WT
KO
H
Relative expression of Pik3cd
PBS 4T1EXO
PBS 4T1EXO
WT
KO
I
NMuMG-WT
NMuMG-KO
+4T1EXO (d)
0 1 3 6 9 11
0 1 3 6 9 11
CREPT
p-AKT
AKT
p-p65
p65
Actin

Figure S6, Related to Figure 6. CREPT-mediated TNFR2 signaling promotes field cancerization induced by CDEs. (A) The volcano plot of the differentially expressed genes in NMuMG cells treated with 4T1EXO compared with untreated NMuMG cells. The blue dots show the significantly (fold change < 0.5, p-value < 0.001) down-regulated genes and the red dots show the significantly (fold change > 2, p-value < 0.001) up-regulated genes. (B) KEGG analysis of the differentially expressed genes between untreated NMuMG cells and NMuMG cells treated with 4T1EXO for 2 weeks. The input gene number, p-value and rich factor of each signaling pathway are indicated. (C) The verification of the inflammation-related-mRNA levels of TNF, NOD2, CXCL1 and CSF1 by qPCR in NMuMG cells treated with 4T1EXO on day 0, 3, 7 and 16. (D) The Venn diagram shows the differentially expressed gene number in NMuMG WT/ KO cells treated with/ without 4T1EXO. (E) KEGG analysis of genes that were significantly upregulated in NMuMG CREPT-WT cells but not significantly upregulated in NMuMG CREPT-KO cells. The input gene number, p-value and rich factor of each signaling pathway are indicated. (F) Heatmap of the mRNA levels of genes related to TNF signaling pathway in NMuMG CREPT-WT and CREPT-KO cells treated with PBS or 4T1EXO. The mRNA levels of Tnfrsf1b (G) and Pik3cd (H) was quantified by qPCR of NMuMG WT/ KO cells treated with/without 4T1EXO. (I) Western blot of CREPT, p-AKT, AKT, p-p65 and p65 in protein extracts of NMuMG CREPT-WT and CREPT-KO cells treated with 4T1EXO for 0, 1, 3, 6, 9 and 11 days.

Table S1. The clinical characteristics of tumors used in PDX model

	Age	type	TNM	ER/PR/ HER-2
Patient 1	61	invasive ductal carcinoma	T2N0M0	-/-/-
Patient 2	60	invasive ductal carcinoma	T2N0M0	-/-/-
Patient 3	63	invasive ductal carcinoma	T3N0M0	-/-/-
